# Macromolecule‐Loaded Hybrid Extracellular Vesicles via Ionic Lipid–Based Post‐Loading for Intracellular Delivery: Functional Evaluation for Neurodegenerative Therapy

**DOI:** 10.1002/jex2.70125

**Published:** 2026-03-17

**Authors:** Lin Cui, Fumiyasu Ono, Tomoko Ichinose, Masahiro Goto, Kohei Ishihama

**Affiliations:** ^1^ NOVIGO Pharma Inc. Fukuoka Japan; ^2^ Department of Applied Chemistry Graduate School of Engineering Kyushu University Fukuoka Japan

**Keywords:** α‐synuclein, antibody therapy, drug delivery system, extracellular vesicles, hybrid extracellular vesicle, nucleic acid therapy, post‐loading

## Abstract

Extracellular vesicles (EVs) are natural carriers that show promise as drug delivery systems (DDS). We developed a non‐invasive post‐loading method to encapsulate macromolecules in EVs using our proprietary ionic lipid base (ILB), which enables electrostatically driver hybridisation without disrupting EV structure. Using EVs isolated from bovine milk and adipocyte‐derived mesenchymal stem cell culture medium, hybrid‐EVs (H‐EV) with different ILB contents encapsulating protein or nucleic acid molecules for delivery were produced in a reproducible manner. The H‐EV retained EV surface markers comparable to those of native EVs after preparation. After cellular uptake, the encapsulated molecules escaped from endosomes into the cytoplasm and exhibited intended functions. In an experiment using SH‐SY5Y neuroblastoma cells in which α‐synuclein (αSyn) aggregation was induced, the introduction of H‐EV encapsulating anti‐αSyn antibodies (Abs) significantly suppressed αSyn aggregation. Furthermore, delivery of anti‐phospho‐AKT Abs using H‐EV promoted caspase 3/7 activity and cell apoptosis. Intravenous administration of H‐EV encapsulating a model Ab into mice resulted in detectable Ab signals in the cerebral cortex, cerebellum, hippocampus and other cells associated with neurodegenerative diseases within 24 h, as assessed by ex vivo imaging. H‐EV enabled loading of various molecules and their targeted transport to specific organs and the cytoplasm, highlighting their potential as a versatile platform for Ab therapies targeting intracellular proteins.

## 1 Introduction

Extracellular vesicles (EVs) are lipid bilayer particles generated and secreted by various cells in the body. As natural delivery carriers, EVs play crucial roles in transporting proteins and nucleic acids between cells and facilitating intercellular communication, and thus have drawn considerable attention over the past decade (Fujita et al. 2018; Matsuzaka and Yashiro 2022; Théry et al. 2018). Unlike artificial carriers such as liposomes and lipid nanoparticles (LNPs), EVs are cell‐derived and offer several advantages, including low immunogenicity, high biocompatibility and the ability to cross biological barriers (Betzer et al. 2020; González et al. 2020; Haney et al. 2015; Liao et al. 2019; Mehryab et al. 2020). Additionally, it has been reported that EVs can cross the blood–brain barrier (BBB) under certain conditions depending on the cell type from which they are secreted. Many researchers are actively investigating the underlying mechanisms for BBB crossing by EVs (Abdelsalam et al. 2023; Banks et al. 2020; Bhatt et al. 2021; Joshi and Zuhorn 2021; Raposo and Stoorvogel 2013; Yang et al. 2021), although these mechanisms are not yet fully understood.

Most conventional drug delivery systems (DDS) require a bottom‐up approach in which the composition of the delivery particle, its components and its targeting specificity are individually developed for each active ingredient. This makes the development of DDS technically demanding and limits the versatility of this technology (Marquez et al. 2024). In contrast, EVs, as naturally assembled cargo carriers, represent a top–down approach for DDS, increasing the flexibility of drug delivery strategies (Ahmad et al. 2022; Chariou et al. 2020; Ebrahimi et al. 2024). The goal of EVs‐based research and an ideal DDS strategy is to realise EVs carrying therapeutic payloads that can be administered through appropriate routes, efficiently delivered to target cells and successfully release their contents to exert intended effects, while preserving EV integrity and functionality.

Drug‐loading approaches for EVs can be broadly classified into pre‐ and post‐loading methods. The pre‐loading method involves genetically modifying EVs‐producing cells to express specific therapeutic molecules, and then isolating and using the EVs secreted by these modified cells (Kosaka et al. 2010; Ohno et al. 2013). The post‐loading method involves isolating EVs secreted by cells and subsequent loading with active compounds using techniques such as electroporation, ultrasonic treatment, extrusion, saponin treatment, freeze–thaw cycles, co‐incubation and polyethylene glycol modification (Ebrahimi et al. 2024, Ohno et al. 2013, Fuhrmann et al. 2015, Zhang et al. 2020), many of which may compromise EV structure or loading efficiency depending on the cargo and conditions.

Compared with preloading strategies, post‐loading enables the introduction of a broad range of molecules into EVs by performing cargo loading after EV isolation. Importantly, because it does not require genetic modification of producer cells—an approach frequently employed in preloading—this method can be readily applied to diverse types of EVs and avoids the risk of unwanted contaminants associated with cell engineering. Moreover, techniques such as electroporation have demonstrated efficient incorporation of hydrophobic drugs and other small molecules that cannot be produced within Cells (Ebrahimi et al. 2024; Zhang et al. 2020; Kooijmans et al. 2013), although such physical or chemical loading procedures may compromise EV integrity or result in variable loading efficiency depending on the cargo.

However, conventional approaches including electroporation, freeze–thaw cycles and ultrasonication have been reported to cause EV membrane damage, aggregation and loss of intrinsic functions thereby limiting their applicability (Ebrahimi et al. 2024; Zhang et al. 2020; Kooijmans et al. 2013). Moreover, in post‐loading methods, only small molecules are typically selected for encapsulation, since large molecules—particularly antibodies (Abs) and nucleic acids—have been especially difficult to load because of their bulky size and strong negative charge.

For example, Abs represent highly specific and low‐toxicity therapeutic molecules (Castelli et al. 2019; Koch and Tew 2023); however, efficient and non‐toxic methods for the in vivo delivery of Abs targeting intracellular antigens have been largely lacking, which remains a major challenge for their clinical application (Koch and Tew 2023; Lv et al. 2020; Wang and Tsourkas 2019).

To overcome these challenges, we developed an ionic lipid base (ILB) platform that shows high fusion compatibility with lipid bilayers (Uddin et al. 2021). Here, we propose a novel post‐loading method that enables the non‐invasive and efficient encapsulation of macromolecules into EVs. This method involves complexing negatively charged therapeutic molecules with ILB to form positively charged particles, which can then efficiently fuse with negatively charged EVs, generating particles referred to as hybrid EVs (H‐EVs).

In this study, to directly confirm the therapeutic efficacy of H‐EV in a disease‐relevant model, an α‐synuclein (αSyn) Ab was used as an in vitro model to target αSyn, a key pathological protein involved in Parkinson's disease. Furthermore, an Ab targeting phosphorylated serine/threonine kinase 1 (AKT, also known as protein kinase B) was used to examine intracellular targeting function of Abs carried by H‐EV.

## 2 Materials and Methods

### 2.1 Reagents

Reagents used for ILB preparation include 1,2‐dimyristoyl‐*sn*‐glycero‐3‐phosphocholine (DMPC) (Avanti Polar Lipids, 850345P).

Reagents used for transmission electron microscopy (TEM) were 4% paraformaldehyde (PFA, Nacalai Tesque, 09514‐85), 1% glutaraldehyde, 2% ammonium molybdate aqueous solution (pH 7.3) and Formvar‐coated PVF‐C10 grid (Okenshoji Co., Ltd., Tokyo, Japan, #10‐1009).

Active Pharmaceutical Ingredient (API) encapsulated in EVs included Human total IgG (Fujifilm Wako, Japan, 149‐09503), anti‐αSyn (SNCA) Ab (for in vitro studies; Sigma–Aldrich, S5566, for in vivo studies: cinpanemab, MedChemExpress, HY‐P99356), Phospho‐AKT1 (pAKT, Ser473) Ab (Thermo Fisher Scientific, Germany, 700392) and FAM3 dye‐labelled pre‐miR negative control (Thermo Fisher Scientific, AM17121).

Abs present on the exterior surface of EVs were digested using proteinase K (Kanto Chemical, Tokyo, Japan, 34060‐96). Encapsulated miRNAs were degraded using RNase A (Thermo Fisher Scientific, EN0531), and Triton X‐100 (Sigma–Aldrich, USA, T8787) was used to solubilise EV membrane.

To investigate intracellular uptake and trafficking pathways, the following inhibitors were used: EIPA (5‐(*N*‐ethyl‐*N*‐isopropyl) amiloride, Cayman Chemicals, USA, 14406), bafilomycin A1 (Funakoshi, Tokyo, Japan, BVT‐0252), wortmannin (Selleck Chemicals, S2758), chloroquine (Selleck Chemicals, S6999), cytochalasin D (Selleck Chemicals, S8184) and chlorpromazine (Fujifilm Wako, Japan, 033‐10581).

For EV labelling, the following fluorescent dyes were used: 3,3′‐dioctadecyloxacarbocyanine perchlorate (DiO, Biotium, #60011), 1,1′‐dioctadecyl‐3,3,3′,3′‐tetramethylindocarbocyanine (Dil, Biotium, #60010), 1,1′‐dioctadecyl‐3,3,3′,3′‐tetramethylindodicarbocyanine perchlorate (DiD, Biotium, #60014) and the ExoGlow‐Vivo EV Labelling Kit (Near IR) (System Biosciences, LLC, USA, EXOGV900A‐1).

A complete list of Abs and additional reagents is provided in Table S1.

### 2.2 Synthesis of ILB

#### 2.2.1 Synthesis of [EDMPC]^+^[TfO]^−^

**FIGURE 1:**
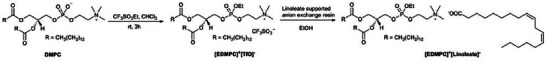
Synthesis of the ionic lipid base (ILB) lipid ([EDMPC]^+^[Linoleate]^−^) in two steps. First, DMPC was converted to [EDMPC]^+^[TfO]^−^. Subsequently, [EDMPC]^+^[TfO]^−^ was transformed into [EDMPC]^+^[Linoleate]^−^. R = CH_3_(CH_2_)_12_.

The synthesis (Figure 1) of [EDMPC]^+^[TfO]^−^ was performed according to the report by Rosenzweig et al. (2000). In a dried 25‐mL two‐necked flask, DMPC (1.36 g, 2.0 mmol, Angene, AGN‐PC‐0QC4OH) was dissolved in chloroform (4 mL). The solution was stirred at room temperature, and then ethyl trifluoromethanesulfonate (258 µL, 2.0 mmol) was added in two portions of 129 µL. After the addition was complete, the reaction mixture was stirred at room temperature for 3 h. The reaction mixture was transferred to a separatory funnel and washed with ethyl acetate (100 mL). Saturated sodium bicarbonate aqueous solution (40 mL) was added, and then the separatory funnel was shaken. The aqueous layer was removed, and the organic layer was washed with saturated saline solution (40 mL). Next, the organic phase was collected and dried over sodium sulfate for 10 min. After filtration to remove the sodium sulfate, the filtrate was concentrated. The crude product was purified by column chromatography using an SI series column (size 60) with chloroform/methanol = 100/0–50/50 (vol/vol) as the eluent. The fraction around a retardation factor (Rf) of 0.34 (developing solvent: chloroform/methanol/water = 70/26/4, vol/vol/vol) was collected, concentrated and vacuum‐dried to obtain the target compound as a white crystalline solid (yield: 1.25 g, 73%). ^1^H NMR (400 MHz, CDCl_3_): *δ* 5.31–5.20 (1H, m), 4.58–4.44 (2H, m), 4.38–4.28 (1H, m), 4.28–4.10 (5H, m), 3.92–3.80 (2H, m), 3.32 (9H, s), 2.31 (4H, dt, *J* = 7.8, 8.7 Hz), 1.68–1.52 (4H, m), 1.47–1.15 (43H, m), 0.88 (6H, t, *J* = 6.9 Hz). HRMS (FAB): *m*/*z* [M − TfO]^+^ Calcd for C_38_H_77_NO_8_P: 706.5387; Found: 706.5390.

#### 2.2.2 Preparation of 10 mM [EDMPC]^+^[Linoleate]^−^ Ethanol Solution

The synthesis was performed with minor modifications to the method reported by Goto et al. (Uddin et al. 2020). An anion‐exchange resin column (20 mL bed volume) was prepared, and a methanol/water (90/10, v/v) solution containing 2% sodium linoleate (200 mL) was slowly passed through the column to load linoleate ions. The column was subsequently washed with methanol/water (90/10 v/v, 400 mL), followed by ethanol (200 mL).

Separately, [EDMPC]^+^[TfO]^−^ (85.6 mg, 0.1 mmol) was dissolved in ethanol (2.5 mL), and the resulting solution was loaded onto the linoleate ion‐exchange resin. Ethanol was used as the eluent. After elution, each fraction was analysed by thin‐layer chromatography (TLC, developing solvent: CHCl_3_/MeOH/H_2_O = 70/26/4, vol/vol/vol; detection reagent: iodine). The fractions containing the target compound were pooled, concentrated to approximately 5 mL, and then diluted with ethanol to a final volume of 10 mL, yielding a 10 mM [EDMPC]^+^[Linoleate]^−^ ethanol solution. ^1^H NMR (600 MHz, CDCl_3_) (Figure S1): *δ* 5.42–5.20 (5H, m), 4.57–4.47 (2H, br), 4.37–4.29 (1H, m), 4.26–4.10 (5H, m), 4.01–3.91 (2H, m), 2.77 (2H, t, *J* = 6.9 Hz), 2.38–2.26 (4H, m), 2.19–2.11 (2H, m), 2.05 (4H, quin, *J* = 6.9 Hz), 1.66–1.53 (6H, m), 0.94–0.83 (9H, m).

### 2.3 Cell Lines

Human cervical cancer cell line (HeLa, ATCC) and human pancreatic cancer cell line (SUIT‐2, ATCC) were cultured in Dulbecco’ Modified Eagle Medium (DMEM; Nacalai Tesque) supplemented with 10% foetal bovine serum (FBS: Invitrogen) and 1% penicillin and streptomycin (P/S, Nacalai Tesque) at 37°C in a humidified incubator with 5% CO_2_. HeLa and SUIT‐2 cells used in experiments were maintained within Passage 10 (P10). Human adipose‐derived mesenchymal stem cells (hADSCs; KAC Inc.) were cultured in α‐minimum essential medium (α‐MEM) supplemented with 10% FBS, 1% P/S and basic fibroblast growth factor (bFGF, 2 ng/mL) at 37°C under 5% CO_2_. hADSCs within Passage 5 (P5) were used for EVs isolation. Human neuroblastoma cell line (SH‐SY5Y; KAC Inc.) was cultured in SH‐SY5Y‐specific medium (DSGM301; KAC Inc.) at 37°C under 5% CO_2_ and was used for the αSyn aggregation assay.

### 2.4 EVs Separation

#### 2.4.1 . Milk‐EVs Separation

Milk‐derived EVs (milk‐EVs) were separated from pasteurised bovine milk. To remove fat globules, casein, cell debris and somatic cells, the milk was centrifuged twice at 3,400 × *g* for 30 min at 4°C. Then, 1% of the supernatant volume of acetic acid (99.7%) was added, followed by centrifugation at 5,000 ×*g* for 20 min at 4°C to separate fat and casein. To remove residual casein, the supernatant was centrifuged at 20,000 × *g* and 4°C for 70 min, followed by filtration through a 0.22‐µm filter. The filtered solution was concentrated using a 100‐kDa filter, and then the concentrate was loaded on a size‐exclusion chromatography column (Meiwafosis Co., Ltd. Gen2 qEV original/35). Twelve 400‐µL fractions of phosphate‐buffered saline (PBS) solution were eluted from the column. Nanoparticle tracking analysis (NTA) and dynamic light scattering (DLS) measurements confirmed that appropriate size particles populations were present in Fractions Fr2–Fr5.

#### 2.4.2 hADSC‐Derived EVs (MSC‐EVs) Separation

hADSCs were seeded at a density of 3×10^6^ cells in 225‐cm^2^ flask (Sigma–Aldrich) and cultured in standard serum‐containing medium until they reached 70%–80% confluency. After two washes with PBS, the medium was replaced with serum‐free medium, and cells were cultured for an additional 72 h. The conditioned medium was then collected for EVs separation. hADSCs from Passage P3 to P5 were used for EVs production. The culture medium from 5 to 10 flasks was sequentially centrifuged at 300 ×*g* for 10 min at 4°C to remove dead cells, 2,000 ×*g* for 15 min at 4°C to remove additional dead cells, and then 10,000 × *g* for 30 min at 4°C to remove cell debris. The supernatant was then filtered through a 0.22‐µm filter and concentrated using a 100‐kDa molecular weight cutoff filter. The concentrated medium was applied to a Gen2 qEV size‐exclusion chromatography column, and 12 fractions (400 µL each) were eluted with PBS solution. NTA and DLS confirmed that particles with appropriate EV size distributions were enriched in Fractions Fr2–Fr5. To assess potential contamination by lipid droplets derived from MSCs, Lipi‐Green dye (Dojindo, LD02) was used. MSCs cultured with or without oleic acid treatment, as well as isolated MSC‐EVs, were stained and analysed by fluorescence microscopy (Figure S2a) and a plate reader (Figure S2b). As shown in Figure S2, abundant lipid droplets were observed in oleic acid‐treated MSCs, whereas only weak fluorescence was detected in untreated MSCs. No Lipi‐Green fluorescence was detected in MSC‐EVs.

### 2.5 H‐EV Production

The preparation of H‐EVs was conducted at room temperature in a clean bench. The ILB ethanol solution and the API (Ab or miRNA) aqueous solution were rapidly mixed at an appropriate ratio, followed by vortexing for 60 s to prepare the primary particles. The primary particles were allowed to stabilise at room temperature for 15 min. After stabilisation, a PBS solution of EVs was added to the primary particles and gently mixed to prepare H‐EVs; the amount of EVs added was determined based on the API quantity. The mixing ratio was determined after validation as shown in Figure S3; however, we regret that the specific ratio cannot be disclosed in this study because it is proprietary information. When determining the mixing ratio, the primary particles were required to carry a positive charge to enable binding to negatively charged EVs. In addition, the resulting H‐EVs needed to exhibit a negative surface charge. The formulation ratio was finalised after confirming by flow cytometry (FCM) analysis that the EVs carried the encapsulated materials.

### 2.6 Animals

For biodistribution study, 8‐week‐old female BALB/cAJcl mice (Kyudo Co., Ltd., Tosu, Japan) weighing 20–25 g were used. All animal experiments were conducted in accordance with protocol approved by the Animal Care and Use Committee of Kyushu University.

For toxicity study, both male and female mice were used to account for potential sex‐dependent differences. Specifically, 8‐week‐old BALB/cAJcl mice were used, with males weighing 23–27 g and females weighing 20–25 g. Toxicity studies were conducted in compliance with protocols approved by the ethics committee of Kyudo Co., Ltd.

### 2.7 Transmission Electron Microscopy (TEM)

The purified EVs were fixed with 4% PFA solution and placed onto hydrophilised formvar‐coated PVF‐C10 grid (Okenshoji Co., Ltd., #10‐1009). After washing with PBS, the samples were fixed with 1% glutaraldehyde for 5 min. Following four washes with ultrapure water, negative staining was performed using 2% phosphotungstic acid aqueous solution. After two additional washes with ultrapure water, the grids were air‐dried overnight and imaged using a transmission electron microscope (JEM‐1400plus, JEOL Ltd., Tokyo, Japan) operated at an accelerating voltage of 80 kV.

### 2.8 Nanoparticle Tracking Analysis (NTA) and Dynamic Light Scattering (DLS)

EV particles were diluted to 1:500 in PBS to a final volume of 1 mL and analysed using a NanoSight NS300 instrument (Malvern Panalytical Ltd., Malvern, UK). NTA measurements were performed according to the manufacturer's instructions (NanoSight NS300 User Manual, MAN0541‐01‐EN‐00, 2017). For each sample, five videos of 60 s duration were recorded at a controlled temperature of 25°C with a syringe pump flow rate of 40 µL/s. Recorded videos were analysed using NanoSight Software version NTA3.3.301 (Malvern Panalytical Ltd.) with a detection threshold set to 5.

Particle size distribution and surface charge (zeta potential) were further characterised by DLS and zeta potential analysis using a Zetasiser Pro (Malvern Panalytical Ltd.). Measurements were performed under identical conditions for all samples. These analyses were used to evaluate particle size, surface charge and the colloidal stability of H‐EVs.

### 2.9 EVs Labelling

EVs were labelled with the lipophilic fluorescent dyes DiD, DiI or DiO. EV labelling was performed based on a previously reported protocol (Cha et al. 2023) with minor modifications. Because DiD, DiI and DiO are prone to aggregation in PBS containing 150 mM NaCl, the EV suspension in PBS was first diluted with ultrapure water to adjust the final NaCl concentration to 20 mM prior to staining. Fluorescent dyes were added to the diluted EV suspension at a final concentration of 2 µM, followed by incubation at 37°C for 30 min. After labelling, free dye was induced to aggregate by adding 10× PBS to restore the solution to 1× PBS, followed by incubation at room temperature for 5 min. Aggregated dye was removed by filtration through a 0.22‐µm syringe filter. The labelled EVs were subsequently concentrated using a 100 kDa Amicon Ultra centrifugal filter unit and used for downstream experiments.

### 2.10 Flow Cytometry (FCM) Analysis of EVs and H‐EV

FCM analysis of EVs was performed using an Attune NxT acoustic focusing cytometer (Thermo Fisher Scientific) equipped with a small particle side‐scatter filter (488/10; Cat. No. 00083194, Invitrogen, Thermo Fisher Scientific). Instrument setup, gating strategy and acquisition parameters were configured in accordance with the manufacturer's guidelines for small particle analysis (Thermo Fisher Scientific) (Lugo‐Gavidia et al. 2021).

FCM was used to evaluate Ab loading into EVs. EVs were analysed without fluorescent labelling. Abs were conjugated with Alexa Fluor 647, and free dye was removed before use as described in Section 2.9. H‐EVs were generated by mixing Alexa Fluor 647–labelled Abs and FAM‐labelled miRNA with EVs under the indicated conditions.

For FCM analysis, samples were diluted in 0.1‐µm–filtered PBS to achieve an appropriate event rate and to minimise swarm detection. Fluorescence‐triggered acquisition based on Alexa Fluor 647 signals was employed to detect Ab‐associated EVs. Unmodified EVs and Alexa Fluor 647–labelled Abs alone were used as negative and background controls, respectively.

The proportion of Alexa Fluor 647‐positive events was quantified as an indicator of Ab loading efficiency into EVs. All samples were analysed under identical instrument settings, and data analysis was performed using Attune NxT Software (Thermo Fisher Scientific).

### 2.11 Western Blot Analysis

Proteins were extracted from whey, milk‐EVs, MSC‐cells, MSC‐EVs and H‐EV using a PRO‐PREP Protein Extraction Kit (Life Science). Protein concentrations were measured using a bicinchoninic acid (BCA) assay kit (Thermo Fisher Scientific). The concentration of extracted proteins was adjusted with sample and loading buffers to ensure 20 µg per lane. Proteins were separated on a 4%–12% Bis‐Tris gel (Invitrogen) and transferred onto a 0.2‐µm polyvinylidene difluoride membrane (Bio‐Rad, USA). The membrane was blocked with Blocking One (Nacalai Tesque) and incubated with the primary Abs specified in Section 2.1. After washing with Tris‐buffered saline containing 0.1% Tween 20, the membrane was incubated with the respective secondary Abs and scanned using a ChemiDoc Imaging System (Bio‐Rad).

### 2.12 Nanoimager and Nikon Microscope

Fluorescently labelled H‐EVs were imaged using a Nanoimager (Oxford Nanoimaging Ltd., UK). EVs were labelled with the lipophilic dye DiI purified by filtration through a 100 kDa Amicon Ultra centrifugal filter to remove free dye. ILB was labelled with TopFluor and purified using a chromatography column, while Abs were labelled with Alexa Fluor 647, and free dye was removed using a PD SpinTrap G‐25 (Cytiva, USA, 28918007) column. The labelled ILB, Abs and EVs were then mixed to generate H‐EVs and observed using a Nanoimager. Images of TopFluor and Alexa Fluor 647 were acquired first, followed by DiI imaging, and the images were overlaid.

For cellular fluorescence imaging, live cells were stained with Hoechst 33342 (Fujifilm Wako, Japan, 346‐07951), and fixed cells were stained with 4′,6‐diamidino‐2‐phenylindole solution (DAPI: Nacalai Tesque, Inc Kyoto, Japan, 19178‐91) to visualize nuclei. Imaging was performed using an N‐SPARC (Nikon) and Nikon‐Ti2 system. Cells were fixed with 4% PFA. Mouse brain tissues were embedded in optimal cutting temperature (OCT) compound (Sakura Finetek USA, Inc.), cryosectioned and fixed with 4% PFA. Fluorescent Nissl staining was performed following the standard protocol using NeuroTrace 500/525 green‐fluorescent Nissl stain (Invitrogen).

### 2.13 Förster Resonance Energy Transfer (FRET)‐Based Lipid Fusion Analysis

ILBs were prepared at a lipid concentration of 0.2 mM and labelled with fluorescent lipids at a final concentration of 0.5 mol% each of NBD‐PE (Avanti Polar Lipids, USA, 810145C) and Lissamine Rhodamine‐PE (Avanti Polar Lipids, 810150P). Lipid fusion was evaluated using an FRET‐based assay by monitoring energy transfer from the donor fluorophore NBD to the acceptor fluorophore Lissamine Rhodamine (40410169).

The reaction was conducted at room temperature for 1 h. Fluorescence intensity was measured as an endpoint using a microplate reader (excitation at 460 nm and emission at 580 nm) in black 96‐well microplates. All samples were prepared in Milli‐Q water. H‐EV samples and ILB controls were measured under identical experimental conditions. All measurements were performed in triplicate (*n* = 3).

### 2.14 αSyn Aggregation Assay

The formation of αSyn aggregates was induced using an αSyn Aggregation Assay Kit (Cosmo Bio Co., Ltd). SH‐SY5Y cells were cotransfected with the pCMV‐SNCA plasmid DNA and F‐αSyn protein. Four hours after transfection, the culture medium was replaced, and H‐EVs were added to the medium. After 12 h, the medium was replaced with fresh medium containing 10% FBS. Forty‐eight hours after the addition of H‐EV, cells were stained following the protocol of the Amyloid Fluorescent Staining Kit to visualise aggregates. The stained aggregates were imaged and analysed using a fluorescence microscope (Nikon‐Ti2 system).

### 2.15 Caspase 3/7 Activity Assay

To evaluate whether the pAKT Ab (Invitrogen), once delivered into the cytoplasm, can bind to AKT protein and regulate cellular function, this Ab was encapsulated in EVs using ILB and introduced into SUIT‐2 pancreatic cancer cells. After 4 h, the culture medium was replaced with FBS‐containing medium, and after 24 h, caspase 3/7 activity was assessed using a Caspase‐Glo 3/7 assay reagent kit (Promega). The reagent was added to the medium following the standard protocol of the manufacturer. Luminescence was measured after 1 h using a microplate reader (Infinite 200 Pro, Tecan).

### 2.16 Animal Experiments

To evaluate the in vivo biodistribution of H‐EVs, Alexa Fluor 647–labelled αSyn Ab (10 mg/kg body weight [BW]), near‐infrared (NIR)‐labelled milk‐EVs (10 mg/kg BW) and H‐EVs prepared at the same Ab and EV doses were dissolved in 200 µL of physiological saline and intravenously administered to mice via the tail vein (each group *n* = 5). As negative controls, PBS and free Alexa Fluor 647 and NIR dyes (at amounts equivalent to those used for labelling) were intravenously injected into separate groups of mice.

Twenty‐four hours after injection, mice were euthanised and transcardially perfused with PBS followed by 4% PFA. Major organs, including the liver, kidney, spleen, pancreas, heart, lung and brain, were harvested. For quantitative analysis using IVIS imaging, the same organs were collected from untreated blank mice, and 20 µL of H‐EV solution corresponding to 10% of the injected dose was injected into each organ ex vivo. The fluorescence signals obtained from these organs were used as reference standards to calculate the delivery efficiency to each tissue.

Brain tissues were fixed overnight in 4% PFA, embedded in OCT compound and cryosectioned into 8‐µm‐thick slices using a microtome. Brain sections were stained with Nissl stain according to the manufacturer's protocol, with the staining solution diluted 1:500 before use. Cell nuclei were counterstained with DAPI. The distribution of Abs in brain tissue was observed using a Nikon fluorescence microscope (Nikon Ti2). To identify microglial cells, sections were immunostained with IBA1/AIF1 (E4O4W) XP Rabbit monoclonal Ab diluted 1:50.

### 2.17 CCK‐8 and LDH Assay

To evaluate the cell toxicity of H‐EV, HeLa cells were treated with H‐EV and assessed for CCK‐8 (Cell Counting Kit‐8) and LDH (lactate dehydrogenase) activity. HeLa cells (1×10^4^ cells) were seeded in 96‐well plates and cultured overnight. The following treatments were added to serum‐free medium the next day: non‐treated (NT) with the same solvent as H‐EV, IgG Ab alone, EVs alone and two different formulations of H‐EV (H‐EV1 and H‐EV2). After treatment for 2 h, cell viability and cytotoxicity were measured using a viability/cytotoxicity multiplex assay kit (Dojindo) according to the manufacturer's protocol. Absorbance was measured with a multiplate reader (Infinite200 Pro, Tecan) at 450 nm (CCK‐8) for viability and 490 nm (LDH) for cytotoxicity. Measurements were taken on Day 1 (2 h after treatment) and continued every 24 h until Day 4. The medium was not changed during the measurement period.

### 2.18 Toxicity Experiment in Mice

The toxicity and immunogenicity of H‐EV were evaluated in mice. H‐EVs were intravenously administered as a single dose, and BW changes, haematology tests and blood chemistry tests were performed 72 h post‐administration, along with the measurement of inflammatory cytokines in the serum. Both male and female mice were used in the experiment to eliminate sex differences. As a negative control, an equal volume of saline (200 µL) and an equal amount of EVs (10 mg/kg BW) were used. H‐EVs were prepared with anti‐IgG Ab (10 mg/kg BW), EVs (10 mg/kg BW) and ILB (100 mg/kg BW). Six male and six female mice were used for each group. BW was measured before and 72 h after administration. Blood samples were collected 72 h after administration, and then euthanasia was performed according to ethical guidelines.

The total blood white blood cells (WBCs), red blood cells (RBCs), haemoglobin (Hgb) concentration, haematocrit (HCT), mean corpuscular volume (MCV), mean corpuscular haemoglobin (MCH), mean corpuscular haemoglobin concentration (MCHC) and platelets (PLT) were measured using an animal haematology analyser (pocH‐100iV Diff, Sysmex). The serum levels of aspartate aminotransferase (AST), alanine aminotransferase (ALT), blood urea nitrogen (BUN), creatinine (CRE) and total bilirubin (T‐BIL) were measured using an animal chemistry analyser (DRI‐CHEM NX600V IC, Fujifilm). Whole blood was centrifuged at 1,200 × *g* for 10 min at room temperature. Inflammatory cytokines in the serum were measured using a mouse pro‐inflammatory cytokine multiplex ELISA kit according to the standard protocol. Absorbance at 450 nm was measured with a multiplate reader.

### 2.19 Statistical Analyses

Statistical analyses were performed using JMP Pro software (ver. JMPPro 17.0.0, SAS Institute). All experiments were independently repeated at least three times (*n* = 3), unless otherwise stated. Data obtained from multiple independent experiments were averaged before normalisation. Results are presented as the mean ± standard deviation (SD). Comparisons between two groups were performed using a two‐tailed unpaired Student's *t* test. Comparisons among multiple groups were conducted using one‐way analysis of variance (ANOVA) followed by either Tukey–Kramer's honestly significant difference (HSD) test (α = 0.05) or one‐way ANOVA with Dunnett's test. A *p* value of less than 0.05 was considered statistically significant.

## 3 Results

### 3.1 Purification and Characterisation of EVs

**FIGURE 2:**
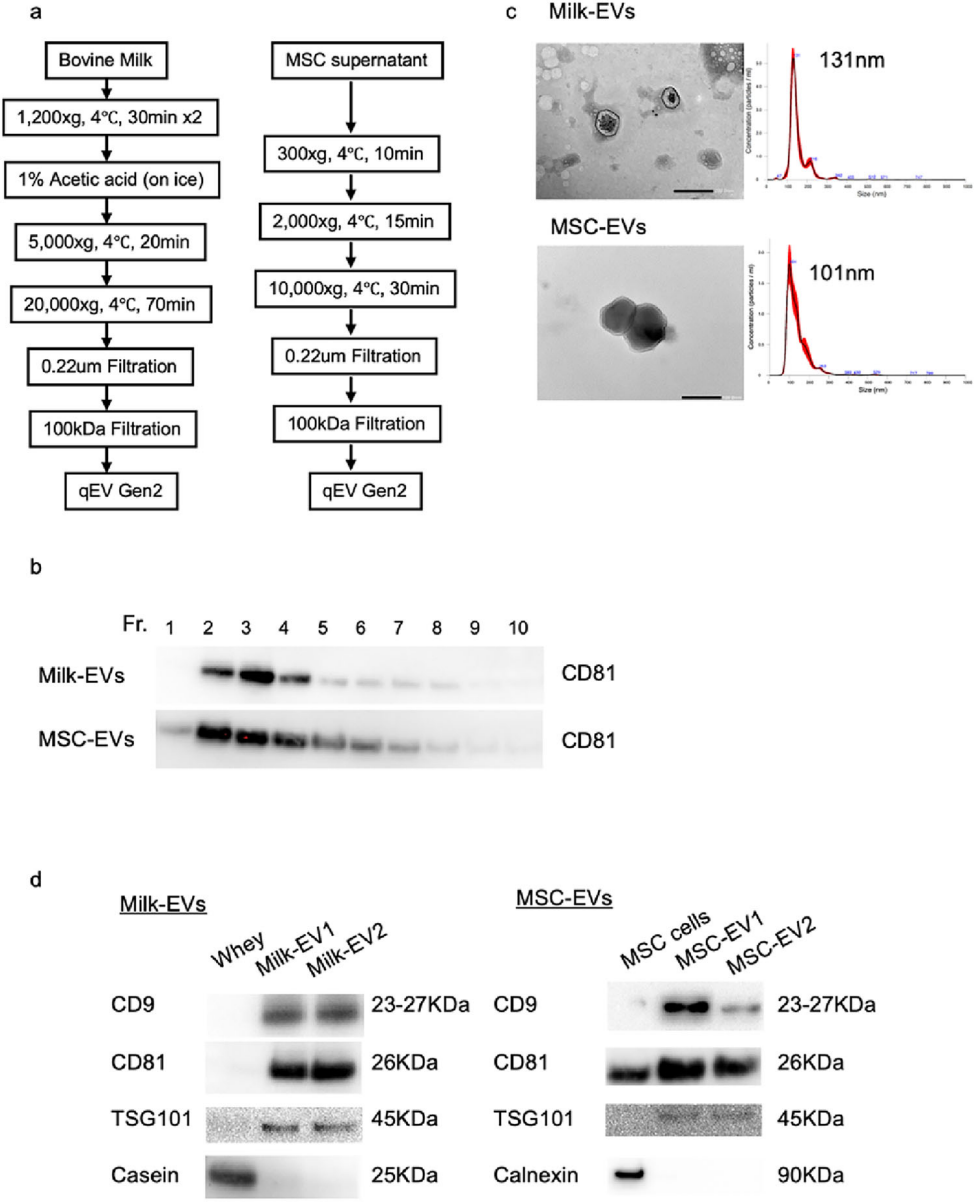
Isolation and characterisation of EVs. (a) Schematic representation of the major steps involved in the isolation of EVs from milk and MSC‐conditioned medium. (b) Western blot (WB) analysis of CD81 protein in Fractions 1–10 obtained after purification and isolation of milk‐EVs and MSC‐EVs using size‐exclusion chromatography with a qEV kit. (c) Transmission electron microscopy (TEM) images of milk‐EVs and MSC‐EVs stained with 2% phosphotungstic acid. The particle size of each EV suspension was measured using a NanoSight microscope after 500‐fold dilution in PBS (scale bar = 200 nm). (d) For milk‐derived samples (left panel), whey, Milk‐EV1 and Milk‐EV2 were analysed for the presence of EV‐enriched tetraspanins CD9 (23–27 kDa) and CD81 (26 kDa), as well as the endosomal marker TSG101 (45 kDa). Casein (25 kDa) was used as a negative control to assess contamination from milk proteins. CD9, CD81 and TSG101 were clearly detected in Milk‐EV fractions, whereas casein was markedly reduced compared with whey, indicating successful enrichment of EVs and removal of soluble milk proteins. For MSC‐derived samples (right panel), MSC cell lysates, MSC‐EV1 and MSC‐EV2 were analysed for EV markers CD9, CD81 and TSG101. The endoplasmic reticulum protein calnexin (90 kDa) was used as a negative marker for cellular contamination. EV, extracellular vesicle; PBS, phosphate‐buffered saline.

In this study, MSC‐EVs and milk‐EVs were used as model EVs. We primarily used milk‐EVs and adipose‐derived MSC‐EVs, which were purified as shown in Figure 2a. The isolation of EVs was confirmed by the presence of the EVs surface marker CD81, detected by western blot (WB) analysis (Figure 2b). EVs Fractions Fr2–Fr4, which showed high expression of CD81, were used in subsequent experiments. The particle number and size in the purified EVs fractions were characterised using NTA and DLS, and the protein concentration was measured using the BCA assay. The particle size of the EVs obtained from approximately 50 mL of milk was 130 ± 10 nm (Figure 2c, upper right), and the particle number was between 1×10^11^ and 4×10^11^ particles/mL. The MSC‐EVs obtained from 100 mL of serum‐free medium in five 225‐cm^2^ flasks (1.5×10^7^ cells) had a particle size of 100 ± 7.8 nm (Figure 2c, lower right) and a particle number between 1×10^9^ and 3.2×10^9^/mL. TEM observation of the purified EVs revealed particles with a lipid bilayer structure and a diameter of approximately 100 nm (Figure 2c, left). WB analysis of purified milk‐EVs isolated from different milk batches with the same protein amount (5 µg) showed strong expression of the EVs markers CD9, CD81 and TSG101, whereas only a weak band was observed in the whey sample (Figure 2d, left). The milk‐derived component casein was removed from the EVs, as confirmed by WB (Figure 2d). MSC‐EVs (EV1 and EV2, which were isolated from MSCs at different passages; the same EVs fraction with 2 µg of protein was used) showed strong expression of the EV markers CD9, CD81 and TSG101 compared with the case for MSCs (2 µg of protein extracted from the MSCs used for isolating MSC‐EV1). The ubiquitous endoplasmic reticulum marker calnexin was removed from the EVs, as confirmed by WB (Figure 2d, right).

### 3.2 Preparation and Properties of H‐EV

**FIGURE 3:**
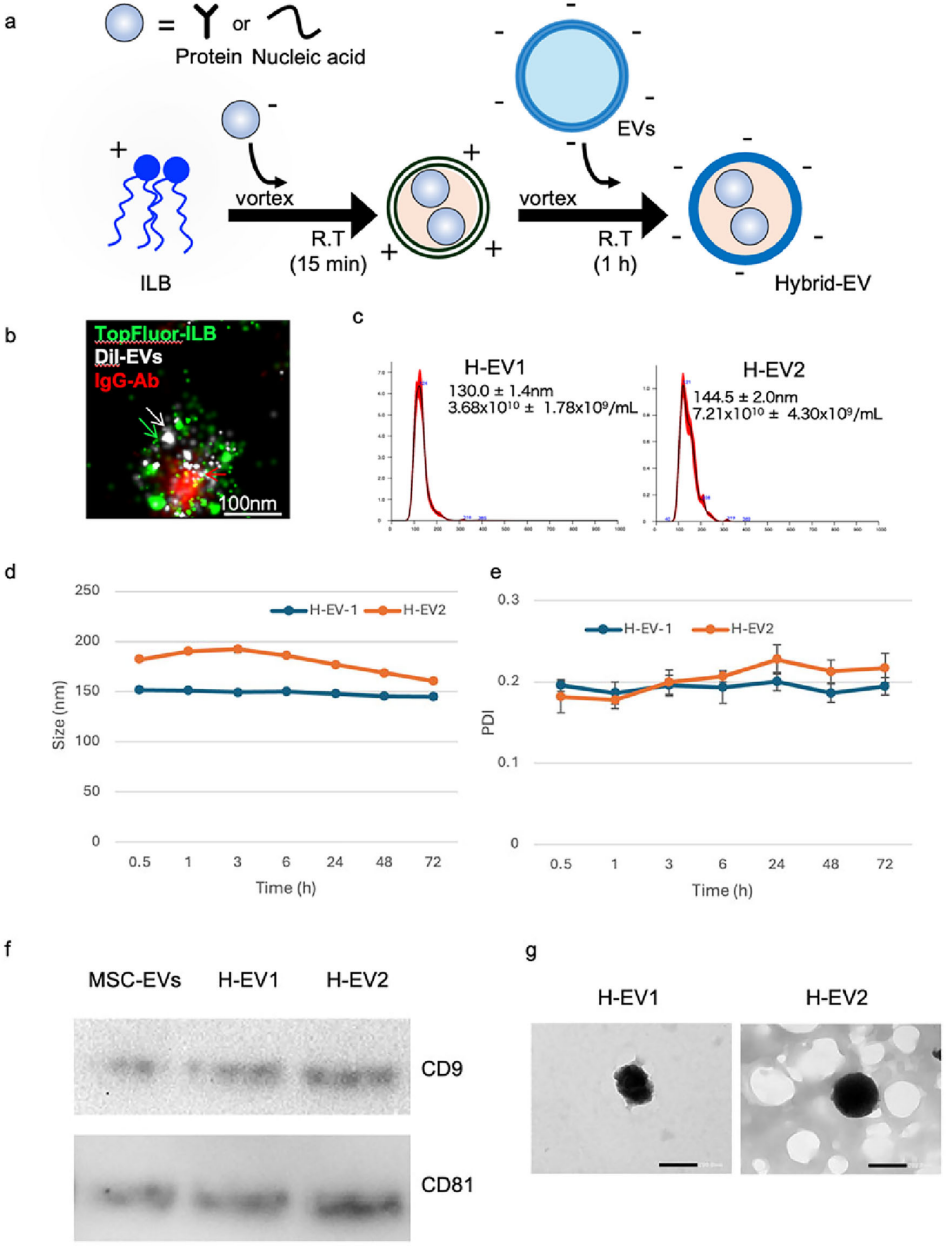
Production of H‐EVs and analysis of antibody or nucleic acid‐loaded H‐EVs. (a) Schematic representation of the production of H‐EV. (b) Localisation of TopFluor‐ILB, DiI‐milk‐EVs and Alexa647 IgG in H‐EV, each conjugated with a different fluorescent dye (scale bar = 100 nm). (c) Particle size (NTA) and particle count of H‐EV. (d, e) DLS measurements showing the change in nanoparticle size over time. (f) WB analysis of the surface markers CD9 and CD81 in EVs, H‐EV1 and H‐EV2. (g) Representative transmission electron microscopy (TEM) images of H‐EV1 and H‐EV2 prepared using different ILB‐to‐cargo ratios. Scale bars, 200 nm. DLS, dynamic light scattering; EV, extracellular vesicle; H‐EV, hybrid‐EV; ILB, ionic lipid base; WB, western blot.

We used ILB synthesised as depicted in Figure 1 to prepare H‐EV (Figure 3a). ILB exhibited a positive charge (24.8 ± 2.9 mV), which allowed electrostatic interaction with biomolecular cargos exhibiting a net negative charge under the experimental conditions used in this study. A mixture of ILB and IgG Abs was vortexed, followed by incubation at room temperature for 15 min to form primary particles. After the primary particles reached charge equilibrium, EVs with a negative charge were added. The ILB and EVs were electrostatically attracted to each other, and after incubation at room temperature for about 1 h, the active ingredients were encapsulated in the H‐EV via membrane fusion.

The optimisation of the mixing ratio among ILB, Abs and EVs is shown in Figure S3. Because native EVs possess a net negative surface charge, ILB–Ab primary particles must acquire a positive charge to enable electrostatic interaction and subsequent hybridisation with EVs. To determine the appropriate ILB content, the ILB ratio was increased stepwise by a factor of two from formulation No. 1 to No. 9. As shown in Figure S3a, the surface charge of the primary particles shifted from negative to positive starting at formulation No. 4, indicating sufficient ILB incorporation to reverse the net charge. Next, primary particles No. 4 to No. 9 were hybridised with EVs at an EV protein amount equivalent to the Ab input, and the surface charge of the resulting H‐EVs was evaluated (Figure S3b). Considering that H‐EVs should retain a net negative surface charge comparable to that of native EVs, formulations No. 4 to No. 6 were identified as optimal. Formulation No. 7 was excluded because its surface charge was close to neutral values. Subsequently, H‐EVs prepared using formulations No. 4 to No. 6 were analysed by FCM to assess Ab encapsulation efficiency (Figure S3c, d). All three formulations exhibited efficient Ab encapsulation, confirming their suitability for H‐EV formation. Based on these results, formulation No. 4 was designated as H‐EV1, and formulation No. 5 was designated as H‐EV2 for subsequent experiments. Although formulation No. 6 showed similar particle size distribution, surface charge and Ab encapsulation efficiency to No. 5, formulation No.5 was selected because it required a lower amount of ILB while maintaining comparable H‐EV characteristics.

To evaluate the robustness and reproducibility of the optimised mixing conditions, H‐EVs were independently prepared using MSC‐EVs derived from three different donors under the same optimised ILB–Ab–EV mixing ratios. As shown in Figure S4, H‐EVs generated from the three MSC‐EV preparations exhibited comparable particle size distributions, polydispersity indices and zeta potentials. These results indicate that the ILB‐mediated hybridisation process yields consistent H‐EV physicochemical properties regardless of donor‐to‐donor variability in MSC‐EVs.

To confirm the fusion between ILB and EVs membranes and localisation of IgG Abs within H‐EVs, the spatial relationship of molecules labelled with different fluorescent dyes was examined using a Nanoimager microscope. As shown in Figure S5, representative images of EVs alone, ILB–Ab primary particles and samples prepared by mixing Abs with EVs are presented. In the H‐EV samples, IgG Abs were predominantly detected within the EV structures, indicating successful incorporation of Abs associated with ILB–EV membrane fusion (Figure 3b). The ratios of ILB, active ingredients and EVs varied depending on the experiment type. For in vitro experiments, two different mixing ratios were used to prepare H‐EV1 and H‐EV2. For in vivo experiments, only one mixing ratio was primarily used, which is referred to as Vivo H‐EV. NTA was performed on H‐EV1 and H‐EV2 (Figure 3c), which gave particle sizes of 130.0 ± 1.4 and 144.5 ± 2.0 nm, respectively. The particle numbers of H‐EV1 and H‐EV2 were 3.68×10^10^ ± 1.78×10^9^ and 7.21×10^10^ ± 4.30×10^9^particles/mL, respectively. The stability of H‐EV was evaluated by DLS measurements. The results showed that the particle size and polydispersity index (PDI) of H‐EV remained stable for 72 h (Figure 3d, e). To further assess long‐term stability, the physicochemical properties of H‐EVs were evaluated for up to 4 weeks under room temperature conditions (Figure S6). Both H‐EV1 and H‐EV2 exhibited minimal changes in particle size distribution throughout the 4‐week period, indicating the absence of aggregation or structural destabilisation. With respect to surface charge, only minor variations in zeta potential were observed, and no substantial changes were detected over time. These findings demonstrate that H‐EVs retain colloidal stability and structural integrity under room temperature storage for extended periods.

To ensure that the H‐EV retained their original EV surface markers, CD9 and CD81 expression were confirmed via WB analysis (Figure 3f). The expression levels of these proteins in H‐EV1 and H‐EV2 were found to be comparable to those in MSC‐EVs, confirming that H‐EV maintained the inherent properties of the original EVs. In addition, TEM analysis revealed that both H‐EV1 and H‐EV2 maintained a typical lipid bilayer structure, with morphology and size comparable to those of naïve EVs, and no apparent aggregation was observed (Figure 3g).

**FIGURE 4:**
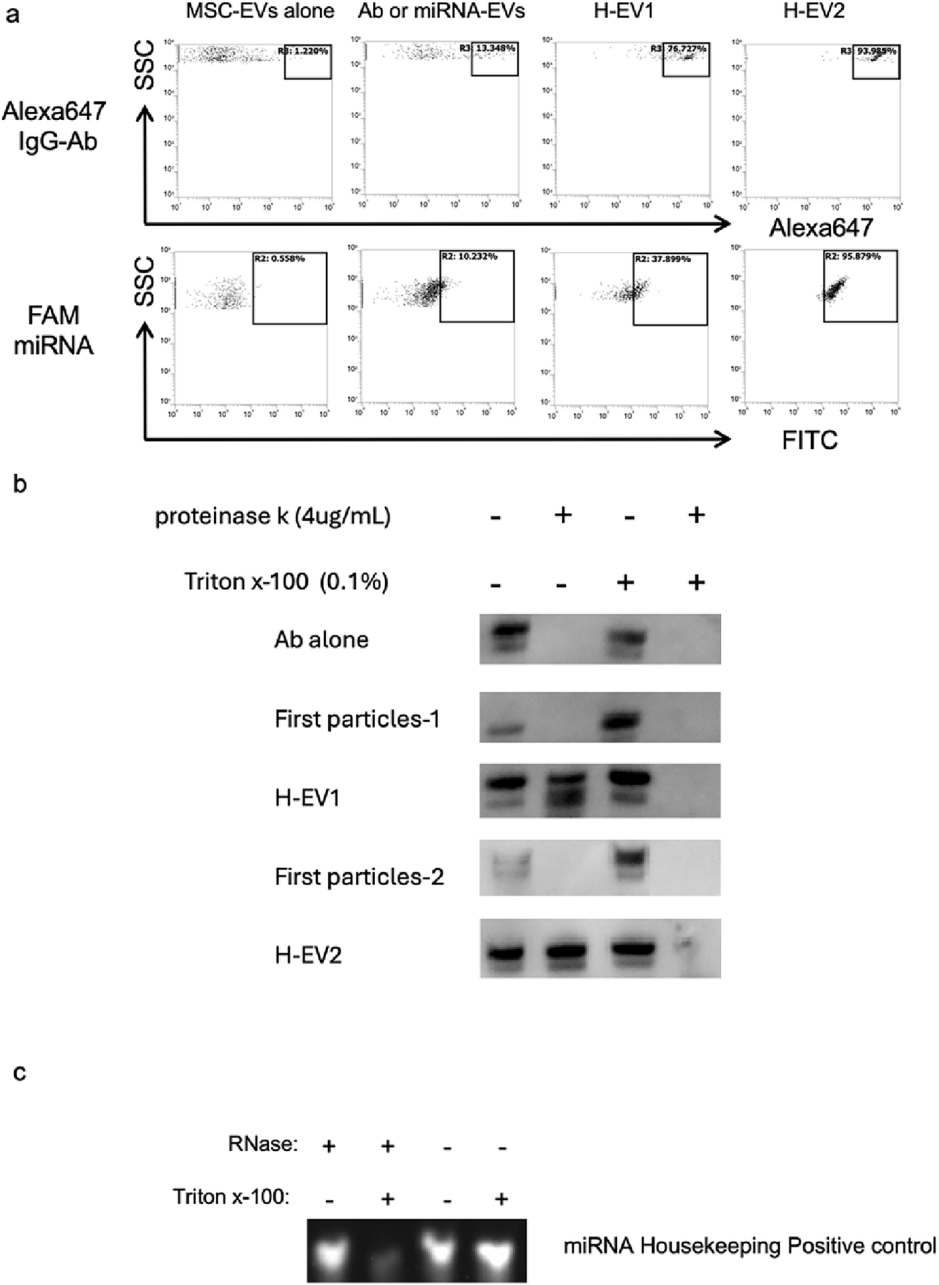
Encapsulation of Abs in EVs. (a) FCM analysis of MSC‐EVs loaded with Alexa647 IgG Abs or FAM3‐labelled pre‐miRNA negative control. Fluorescent signals from Abs and miRNA incorporated into MSC‐EVs were analysed. The loading efficiencies of Abs (upper panel) and miRNA (lower panel) in MSC‐EVs compared with those of MSC‐EVs alone, EVs mixed with IgG Abs or miRNA and H‐EV1 and H‐EV2 are shown. (b) Encapsulation of anti‐α‐synuclein antibody (anti‐αSyn Ab) in milk‐derived H‐EVs was evaluated by western blot analysis. H‐EVs containing 30 ng of anti‐αSyn Ab were incubated with or without proteinase K (4 µg/mL) and Triton X‐100 (0.1%) at 37°C for 1 h, followed by western blotting to detect antibody signals. Proteinase K treatment alone did not markedly reduce the antibody signal, whereas disruption of the EV membrane by Triton X‐100 resulted in substantial signal loss, indicating that the antibodies were protected by the EV lipid bilayer and encapsulated within H‐EVs. (c) Encapsulation of miRNA in milk‐derived H‐EVs was assessed by an RNase protection assay. H‐EVs containing miRNA were incubated with or without RNase (10 ng/mL) and Triton X‐100 (0.1%) at 37°C for 1 h, followed by electrophoresis on a 15% polyacrylamide gel. miRNA signals were preserved after RNase treatment alone but were eliminated upon membrane disruption with Triton X‐100, demonstrating intraluminal localisation of miRNA within H‐EVs. The RNase (−) condition represents the total input miRNA. αSyn, α‐synuclein; Ab, antibody; EV, extracellular vesicle; FCM, flow cytometry; H‐EV, hybrid‐EV.

To verify the encapsulation of Abs in the prepared H‐EV, we performed FCM analysis (Figure 4a). Using a small particle side‐scatter filter (Lugo‐Gavidia et al. 2021) designed for nanoparticle analysis, we identified the EVs population and assessed the percentage of EVs containing Alexa647‐IgG Abs. In the MSC‐EVs‐alone group, we identified an Alexa647‐negative population. For the negative control, which consisted of Alexa647‐IgG Abs mixed with MSC‐EVs, 13.35% of the EV population was positive for Alexa647‐IgG. In contrast, 76.73% of EVs in H‐EV1 and 93.99% of EVs in H‐EV2 contained encapsulated Alexa647‐IgG Abs. Similarly, we confirmed the encapsulation of FAM‐miRNA in MSC‐EVs. Mixing FAM‐miRNA with MSC‐EVs resulted in 10.23% of EVs containing FAM‐miRNA. For H‐EV, the percentage of EVs containing FAM‐miRNA was 37.90% in H‐EV1 and 95.88% in H‐EV2 (Figure 4a, lower panel).

To compare the encapsulation efficiency of our ILB‐based post‐loading method with conventional approaches, H‐EVs were prepared using electroporation (Pomatto et al. 2019), freeze–thaw cycling (Zeng et al. 2023) and simple incubation (Zeng et al. 2023) under standardised conditions. For electroporation, IgG Abs (100 ng) were mixed with MSC‐EVs (100 ng) and processed using the Neon Transfection System (Thermo Fisher Scientific) at 500 V, 20 ms, with two pulses. For the freeze–thaw method, the same amounts of IgG Abs and MSC‐EVs were mixed, incubated at room temperature for 30 min and subjected to three cycles of freezing at −80°C followed by thawing. For the incubation method, IgG Abs and MSC‐EVs were mixed and incubated at 37°C for 1 h. After each loading procedure, samples were treated with proteinase K (4 µg/mL) at 37°C for 30 min to digest non‐encapsulated or surface‐associated Abs. The remaining Ab signal, corresponding to protease‐protected and thus encapsulated IgG, was analysed by western blotting. As shown in Figure S7, the ILB‐based H‐EV preparation resulted in a markedly higher level of protease‐resistant IgG compared with electroporation, freeze–thaw or incubation methods, indicating superior encapsulation efficiency. These results demonstrate that the ILB‐mediated fusion strategy enables more efficient and stable Ab encapsulation within EVs than commonly used post‐loading techniques.

To further investigate whether ILB–cargo primary particles were physically associated with EV membranes rather than merely coexisting with or adsorbing onto the EV surface, FRET analysis was performed using differentially labelled ILB–cargo complexes and EV membranes (Figure S8).  A clear FRET signal was detected in H‐EV samples, whereas no FRET signal was observed in control samples containing ILB–cargo complexes alone. These results indicate that the donor and acceptor fluorophores were in extremely close proximity, within a few nanometers, consistent with nanoscale association between the ILB–cargo complexes and EV membranes. The presence of an FRET signal supports the notion that ILB–cargo primary particles interact intimately with the EV lipid bilayer, suggesting membrane fusion or tight membrane integration rather than simple coexistence or surface adsorption. However, because FRET analysis alone cannot distinguish surface association from intraluminal localisation, protease and nuclease protection assays were subsequently performed to determine whether the cargo molecules were encapsulated within the EVs (Figure 4b, c).

Although FCM analysis confirmed the encapsulation of Abs and miRNA in EVs, it did not provide evidence that the molecules were encapsulated within the lipid bilayer of the EVs. To confirm the encapsulation of Abs or miRNA within the EVs, we conducted WB analysis and electrophoresis using milk‐EVs. Addition of proteinase K, which digests free Abs and those bound to the EV membrane, enabled us to confirm the presence of proteins that were protected within the H‐EV and thus were not digested by proteinase K (Torres et al. 2021). When free anti‐αSyn Abs or ILB‐Ab primary particles (first Particles 1 and 2) were treated with proteinase K, the Ab signal was almost completely abolished, indicating that these Abs were exposed to the external environment (Figure 4b).

In contrast, anti‐αSyn Ab signals in H‐EV1 and H‐EV2 were largely preserved after proteinase K treatment, demonstrating that the Abs were protected by the EV membrane. Subsequent addition of Triton X‐100 to disrupt the EV membrane resulted in a marked reduction of the Ab signal, and proteinase K was then able to completely digest the encapsulated Abs. The anti‐αSyn Ab signal remained detectable in the absence of proteinase K, further supporting that the Abs were enclosed within the EV lumen and shielded by the lipid bilayer.

To confirm the encapsulation of miRNA in EVs, a similar RNase protection assay was performed. When RNase (10 ng/mL) was added, free miRNA was completely degraded. In contrast, miRNA associated with H‐EVs remained intact following RNase treatment, indicating protection by the EV membrane. Upon disruption of the EV membrane with Triton X‐100, RNase was able to digest all miRNA, confirming that the miRNA had been encapsulated within the EVs rather than adsorbed onto their surface (Figure 4c).

### 3.3 Mechanism of Intracellular Uptake of IgG Abs Encapsulated in H‐EV

**FIGURE 5:**
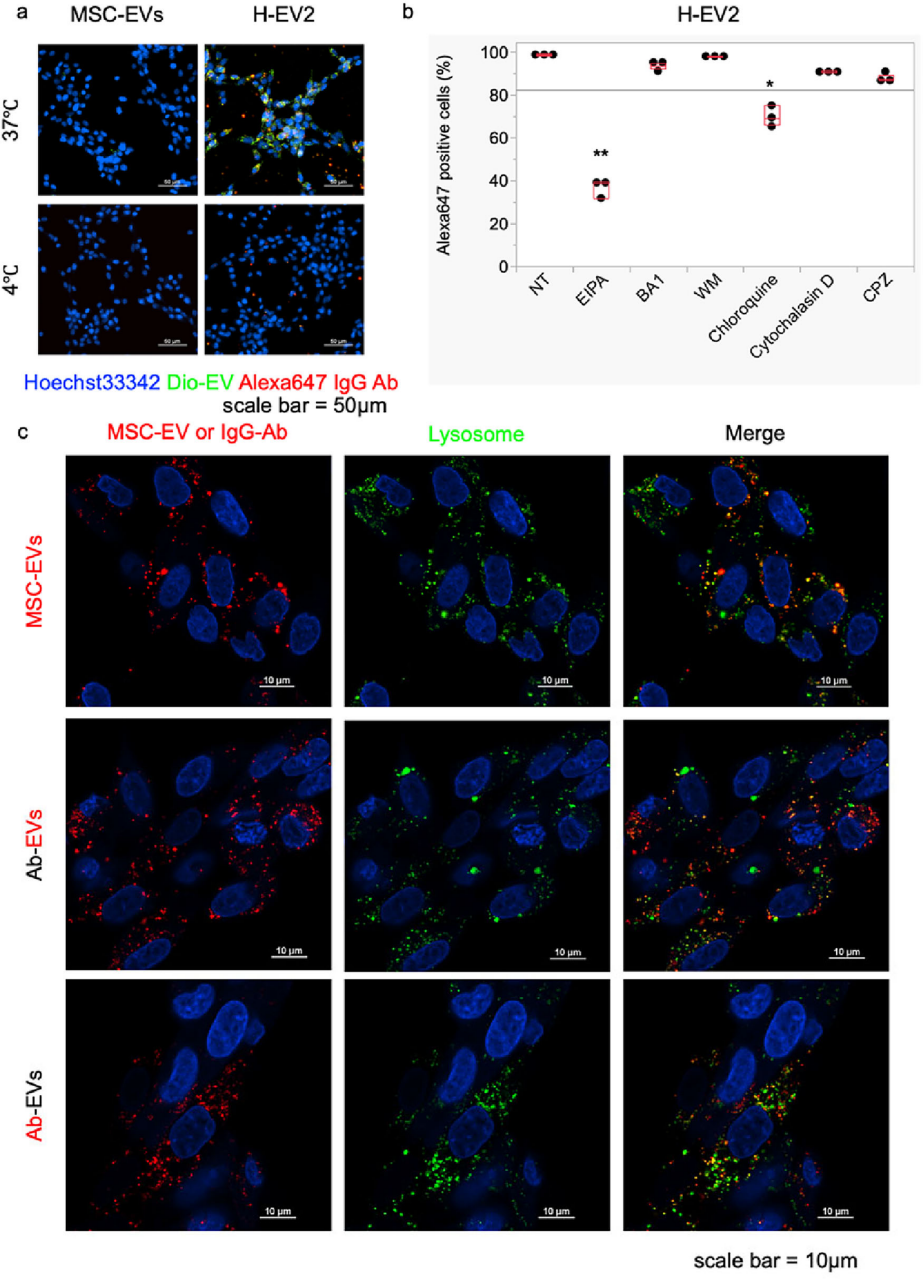
Confirmation of the cellular uptake pathway. (a) Fluorescent images of HeLa cells after incubation with H‐EV alone or H‐EV at 4 and 37°C for 6 h post‐treatment. EVs were stained with DiO and Abs were labelled with Alexa647 (IgG Ab). Scale bar = 50 µm. (b) Flow cytometry (FCM) analysis of HeLa cell uptake of Alexa647‐labelled IgG Abs encapsulated in H‐EV in the presence of endocytosis inhibitors (EIPA: 100 µM, BA1: 0.5 µM, wortmannin: 1 µM, chloroquine: 0.5 µM, cytochalasin D: 2 µM, chlorpromazine: 10 µM). FCM was performed 24 h after transfection. The NT group was treated with DMSO instead of inhibitors. The bar graph shows the percentage of cells with Alexa647 IgG uptake 24 h post‐transfection. Statistical significance was assessed by ANOVA with Dunnett's test compared with the NT group. *n* = 3, mean ± SD, *p < 0.05, **p < 0.01. (c) Upper panel: MSC‐EVs stained with DiD (0.75 µg). Middle panel: MSC‐EVs (0.75 µg) loaded with IgG Abs and stained with DiD. Lower panel: MSC‐EVs loaded with Alexa647 IgG Abs without staining. These MSC‐EVs and H‐EV were added to HeLa cells. After 6 h, the medium was replaced with 10% FBS, and LysoTracker Red DND‐99 (200 nM) was added. After 24 h, cells were fixed with 4% PFA, and nuclei were stained with DAPI. DiD (Ex 645 nm/Em 663 nm), Alexa647 (Ex 650 nm/Em 665 nm), LysoTracker Red DND‐99 (Ex 577 nm/Em 590 nm). The overlap was visualized as yellow in the merged images. Scale bar = 10 µm. Ab, antibody; ANOVA, analysis of variance; EV, extracellular vesicle; FBS, foetal bovine serum; H‐EV, hybrid‐EV; PFA, paraformaldehyde.

To investigate the mechanism by which H‐EVs encapsulating Abs are taken up into cells, we conducted an evaluation using HeLa cells (Figure 5a, b). As shown in Figure 5a, H‐EVs were added to cells cultured at 4℃ and 37°C. After 6 h, intracellular uptake was completely inhibited in cells maintained at 4°C, whereas uptake was observed in cells at 37°C. This suggests that H‐EV enter cells via energy‐dependent endocytosis rather than membrane fusion. To determine the specific pathway involved in the intracellular uptake, we used recipient cells and added various inhibitors targeting different uptake pathways. After pre‐incubating the cells with different inhibitors for 1 h, H‐EVs were added, and the cells were cultured in serum‐free medium for 24 h. FCM analysis was performed to assess the percentage of cells that had taken up the encapsulated Abs and reveal the effect of different inhibitors on intracellular uptake (Figure 5b). The inhibitors used were EIPA (an Na^+^/H^+^ inhibitor that blocks macropinocytosis), wortmannin (a PI3K inhibitor), chlorpromazine (an inhibitor of clathrin‐dependent endocytosis), cytochalasin D (an actin polymerisation inhibitor), bafilomycin A1 (blocks the transition to late endosomes and lysosomes) and chloroquine (inhibits lysosomal acidification). The concentrations used were optimised based on preliminary screening and were set below concentrations that are toxic to cells. Among these inhibitors, EIPA and chloroquine strongly suppressed intracellular uptake of the H‐EV‐encapsulated Abs, indicating that H‐EVs are primarily taken up via macropinocytosis and escape from endosomes under the acidic conditions of lysosomes. Statistical analysis was conducted by confirming normality and then performing one‐way ANOVA with Dunnett's test (*α* = 0.05) for comparisons with the NT control.

Next, we investigated the intracellular localisation of IgG Abs encapsulated in MSC‐EVs in HeLa cells after incubation for 24 h. Lysosome, Ab and H‐EV localisation were observed using confocal microscopy (Figure 5c). For this experiment, H‐EVs were prepared using either Alexa647‐labelled Abs and DiD‐labelled MSC‐EVs or unlabelled IgG Abs and MSC‐EVs. LysoTracker Red DNS‐99 signals were pseudo‐coloured green to distinguish them from EV and Ab fluorescence. Initially, when DiD‐labelled EVs were used, colocalisation with lysosomes was observed after 24 h, with some EVs found in lysosomes and a considerable fraction of EVs were observed outside lysosomes (Figure 5c, upper panel). These observations are consistent with previous studies demonstrating that EVs enter cells via endocytosis and escape from endosomes (Nakase and Takatani‐Nakase 2022; Tian et al. 2014).

When unlabelled IgG Abs were encapsulated in labelled MSC‐EVs and the localisation of H‐EV relative to lysosomes was observed, a higher intracellular uptake of H‐EV was noted compared with that of EVs alone. Additionally, some H‐EV were found outside lysosomes, suggesting partial escape from lysosomal compartments (Figure 5c, middle panel).

Finally, when unlabelled MSC‐EVs encapsulating Alexa647‐IgG Abs were examined for colocalisation with lysosomes (Figure 5c, lower panel), a lower number of Alexa647‐IgG were detected compared with that under the previous conditions, colocalisation with lysosomes decreased, compared to EVs alone, H‐EV had more EVs that were not bound to lysosomes and were free in the cytoplasm. Also, like EVs, there was more Alexa647 IgG Ab in H‐EV that was free in the cytoplasm and not bound to lysosomes. This experiment was conducted using unlabelled MSC‐EVs (Figure 5c, lower panel) and assuming the same movement as that of the labelled MSC‐EVs (Figure 5c, middle panel). These results suggest that EVs encapsulating IgG Abs are internalised via macropinocytosis and release their Ab cargo in to the cytoplasm during the endosomal maturation process before lysosomal degradation. As shown in Figure S9, neither DiD nor Alexa Fluor 647 fluorescence was detected inside cells when the dyes were administered alone or in combination with ILB, indicating that the fluorescent signals observed in H‐EV‐treated cells were not derived from free dyes or ILB–dye complexes.

### 3.4 Functional Evaluation of Abs Delivered to the Cytosol

**FIGURE 6:**
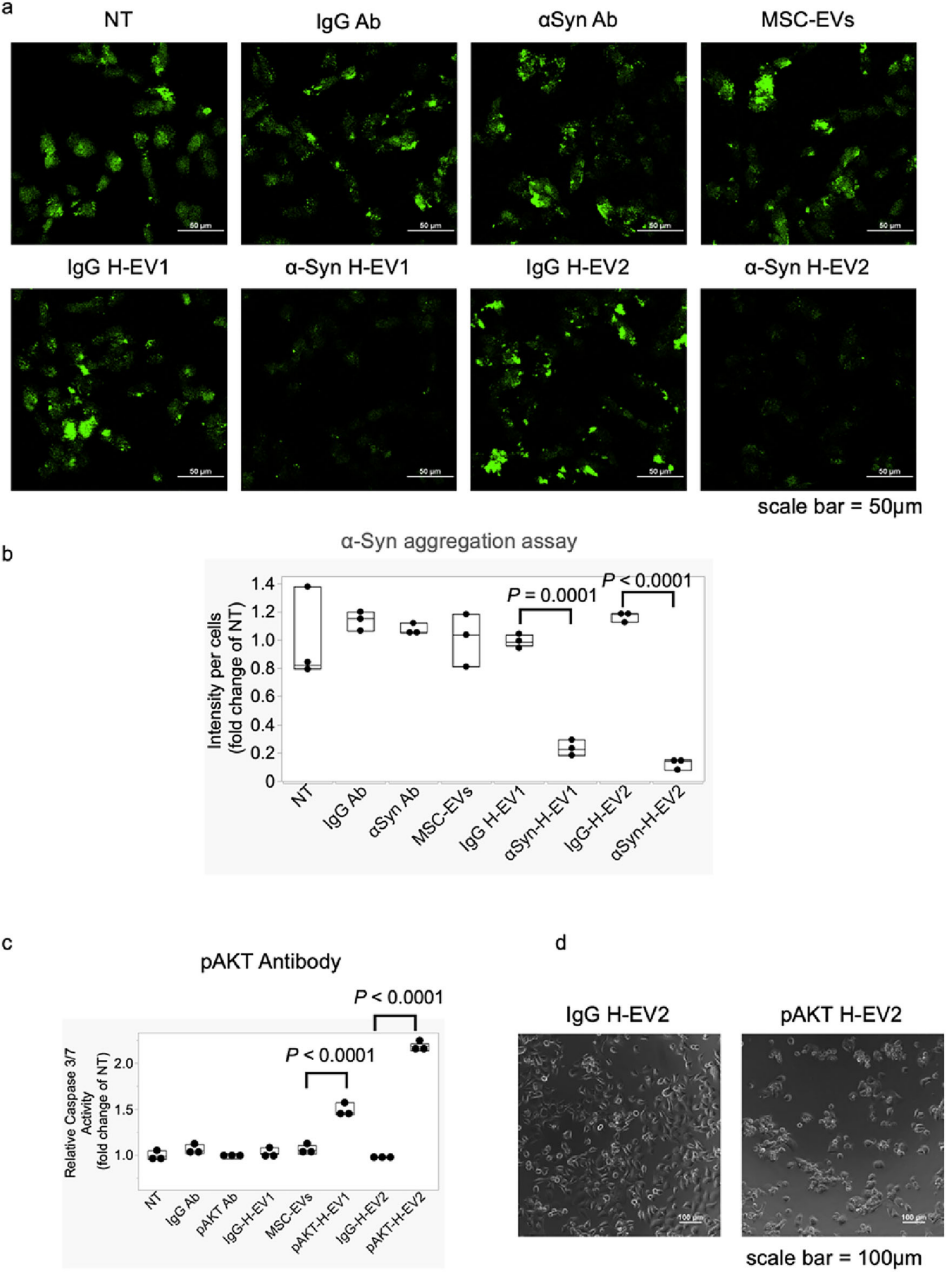
Functional confirmation of Ab‐loaded H‐EV. (a, b) αSyn‐H‐EV1 and αSyn‐H‐EV2, as well as control   IgG‐loaded H‐EV, were evaluated for their ability to suppress αSyn aggregation. SH‐SY5Y cells were cotransfected with pCMV‐*SNCA* DNA and F‐αSyn seeds. After 4 h, the medium was replaced, and H‐EV particles were added. After 48 h, αSyn aggregation was visualised. (b) Relativefluorescence intensity of aggregates per cell compared with NT controls. Statistical analysis was performed using Student's *t* test compared with the corresponding IgG group (*n* = 3, mean ± SD). scale bar = 50 µm. (c, d) Effect of pAKT ab‐loaded H‐EV on caspase 3/7 activity in SUIT2 cells. Statistical analysis was performed using Student's *t* test compared with IgG controls (*n* = 3, mean ± SD). Scale bar = 100 µm. αSyn, α‐synuclein; Ab, antibody; EV, extracellular vesicle; H‐EV, hybrid‐EV; pAKT, phospho‐AKT1.

To evaluate the functionality of Abs delivered into the cytosol, we conducted experiments targeting αSyn (Figure 6a, b). As an in vitro αSyn aggregation model, we cotransfected SN‐SY5Y neuroblastoma cells with pCMV‐*SNCA* DNA and fibrillised αSyn protein using a cotransfection kit. After 4 h, H‐EV (derived from MSCs) encapsulating 1 µg of αSyn Abs were added. After 48 h, αSyn aggregation was visualised using an amyloid fluorescent staining kit. As control groups, we also treated cells with H‐EV encapsulating the same amount of IgG Abs, free αSyn Abs, free IgG Abs and MSC‐EVs alone. As shown in Figure 6a, b, free αSyn Abs, free IgG Abs and MSC‐EVs alone did not inhibit αSyn aggregation. Similarly, the αSyn aggregation levels of samples exposed to IgG H‐EV, regardless of their Ab content, showed no significant difference from that of the NT group. In contrast, αSyn H‐EV1 and αSyn H‐EV2 significantly inhibited (*p* < 0.001) αSyn aggregation compared with that observed in the IgG H‐EV groups. These results suggest that αSyn Abs were successfully delivered into cells via H‐EV and likely interacted with intracellularαSyn, thereby suppressing αSyn aggregation.

Next, we evaluated the functionality of the Abs in an apoptosis‐inducing model targeting AKT, an important protein in cell proliferation. Normally, inhibition of AKT phosphorylation activates apoptosis‐inducing factors such as Bad (Bcl‐2‐associated dominant inhibitor of apoptosis), Bax (Bcl‐2‐associated X protein) and caspase‐9, leading to downstream activation of caspase‐3 and caspase‐7, which cleave proteins and induce apoptosis (Song et al. 2019). To assess whether pAKT Abs delivered into the cytosol can inhibit AKT signalling, we measured caspase‐3/7 activity in SUIT‐2 pancreatic cancer cells. Two formulations of pAKT Ab‐encapsulated H‐EV were prepared, which are denoted as pAKT H‐EV1 and pAKT H‐EV2. As shown in Figure 6c, pAKT H‐EV1 increased caspase‐3/7 activity by 1.5 times (*p* < 0.001) compared with that of the control IgG H‐EV1, whereas pAKT H‐EV2 doubled caspase‐3/7 activity (*p* < 0.0001) compared with IgG H‐EV2. Furthermore, microscopy analysis after 24 h revealed that cells treated with pAKT H‐EV2 possessed a rounded morphology indicative of apoptosis (Figure 6d). This morphological change was not observed for IgG H‐EV2‐treated cells, further confirming that apoptosis was induced by the AKT phosphorylation blockade. These findings demonstrate that H‐EV effectively delivered functional Abs into the cytosol, where the Abs exerted functional intracellular effects, including modulation of AKT signalling and induction of apoptosis.

### 3.5 In Vivo Biodistribution of Vivo H‐EVs and Brain Delivery

**FIGURE 7:**
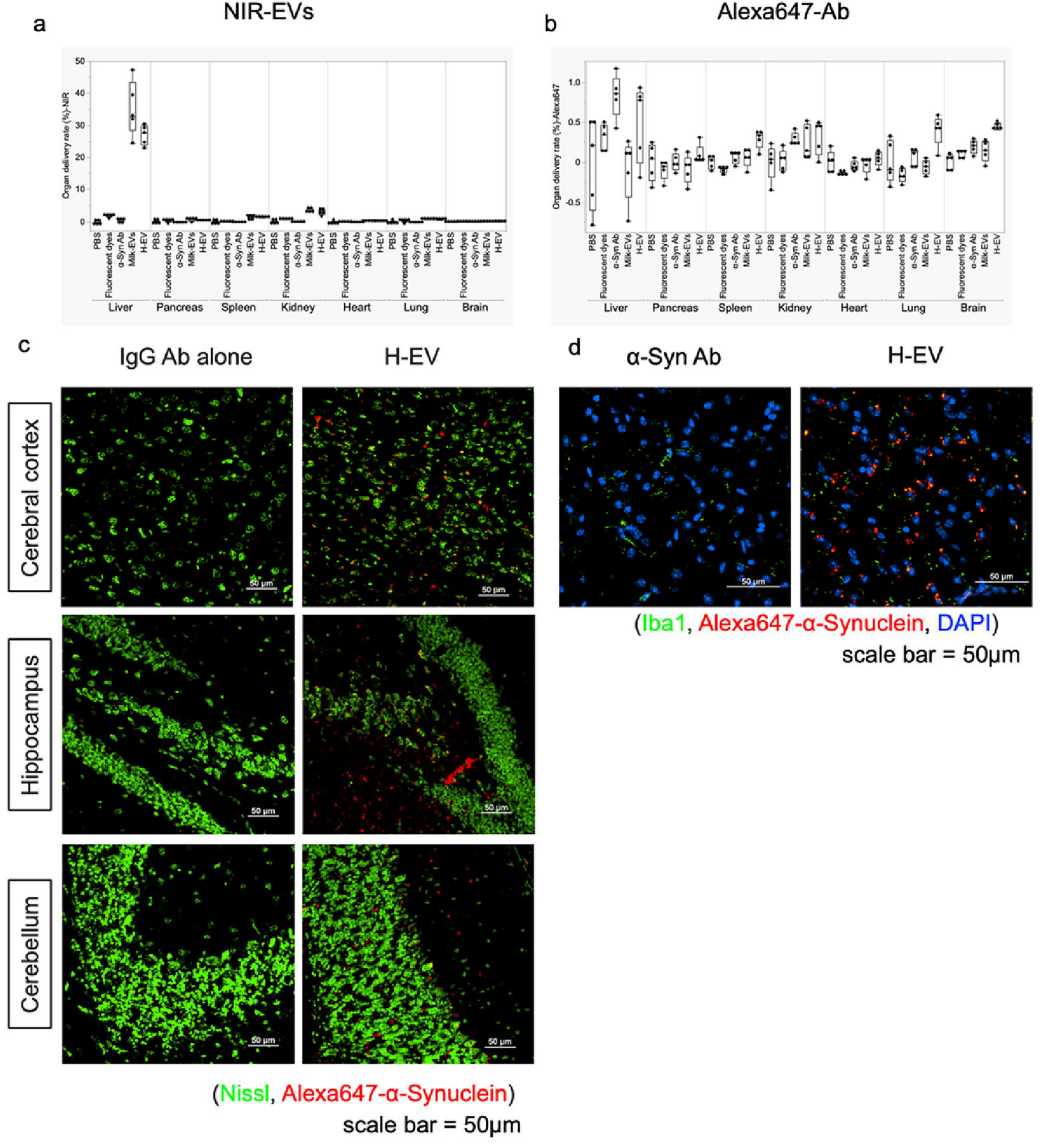
In vivo biodistribution of H‐EV and brain delivery (a) Whole‐organ biodistribution of NIR‐labelled samples analysed by IVIS imaging 24 h after intravenous administration in mice. The percentage of organ delivery was quantified for the liver, pancreas, spleen, kidney, heart, lung and brain. Groups included PBS, fluorescent dye alone, α‐synuclein antibody (α‐Syn Ab), milk‐derived EVs (Milk‐EV) and H‐EV. (b) Quantitative biodistribution of Alexa647‐labelled α‐Syn Ab detected by IVIS imaging 24 h post‐injection. Signals were normalised using a calibration standard corresponding to 10% of the total injected dose for each organ. H‐EVs exhibited enhanced accumulation in the brain compared with free antibody, fluorescent dye alone and Milk‐EV controls. (c) Alexa647‐labelled αSyn Abs alone and Alexa647‐labelled αSyn Ab‐loaded H‐EV were injected intravenously. After 24 h, the brain tissue was harvested following PBS and 4% PFA perfusion, and then 8‐µm‐thick OCT‐embedded cryosections were prepared. Nissl staining was performed to observe the distribution of Alexa647‐labelled αSyn Abs in the brain. Green: Nissl, red: Alexa647‐labelled αSyn Abs. Scale bar = 100 µm. (d) Colocalisation analysis with IBA1 suggested association with microglial cells. Green: IBA1, red: Alexa647‐labelled αSyn Abs, blue: DAPI. Scale bar = 25 µm. αSyn, α‐synuclein; Ab, antibody; DAPI, 4′,6‐diamidino‐2‐phenylindole solution; EV, extracellular vesicle; H‐EV, hybrid‐EV; NIR, near‐infrared; PBS, phosphate‐buffered saline; PFA, paraformaldehyde.

To evaluate the in vivo biodistribution of H‐EVs, milk‐EVs were labelled with an NIR dye and loaded with Alexa Fluor 647–labelled αSyn Ab (200 µg) to generate Vivo‐H‐EVs, which were administered intravenously at a dose of 10 mg/kg BW. Vivo‐H‐EVs were injected into the tail vein of 8‐week‐old mice. Twenty‐four hours after injection, mice were euthanised and perfused with PBS followed by 4% PFA. Major organs, including the liver, kidney, spleen, pancreas, heart, lung and brain, were harvested and subjected to ex vivo fluorescence imaging using an IVIS system. For quantitative analysis, organs from untreated mice were used as backgroud controls, and Vivo‐H‐EVs corresponding to 10% of the injected dose were added to each organ. All samples were placed in the same 24‐well plate and imaged under identical IVIS settings. Representative images are shown in Figure S10, and quantitative analyses are presented in Figure 7a, b. As shown in Figure 7a, the in vivo biodistribution of EVs and H‐EVs at 24 h after intravenous administration, evaluated by NIR fluorescence imaging, revealed predominant accumulation in the liver, kidney and spleen, which is consistent with previous reports on EV biodistribution (Choi et al. 2021). With respect to Ab delivery, H‐EVs exhibited slightly higher accumulation in the spleen and lung compared with free Ab administration. Notably, Ab signals in the brain were more pronounced in the H‐EV group than in any other treatment group, whereas only minimal signals were detected in the brain following administration of Ab alone, fluorescent dyes or native EVs. These results indicate that encapsulation of Abs into H‐EVs enhances their delivery to the brain while maintaining a biodistribution profile largely comparable to that of conventional EVs.

The brains were excised, embedded in OCT compound and cryosectioned into 8‐µm‐thick sections. The localisation of Alexa647‐labelled αSyn Abs was examined by fluorescence microscopy (Figure 7c). To visualise neuronal structures, Nissl fluorescence staining was performed, which revealed that Alexa647‐labelled αSyn Abs were widely distributed in the cerebral cortex, hippocampus and cerebellum. Additionally, to identify microglia, IBA1 staining was conducted, which suggested colocalisation with Alexa647‐labelled αSyn Abs (Figure 7d). A considerable amount of Alexa647‐labelled αSyn Ab uptake was observed in microglia of the cerebral cortex, consistent with a report by Zheng et al. (2017), who showed that EVs that enter the brain are primarily captured by microglia. These findings indicate that αSyn Ab signals were present within brain tissue following administration of in Vivo H‐EV, consistent with enhanced brain‐associated delivery compared with free Ab.

### 3.6 Cytotoxicity of H‐EV

**FIGURE 8:**
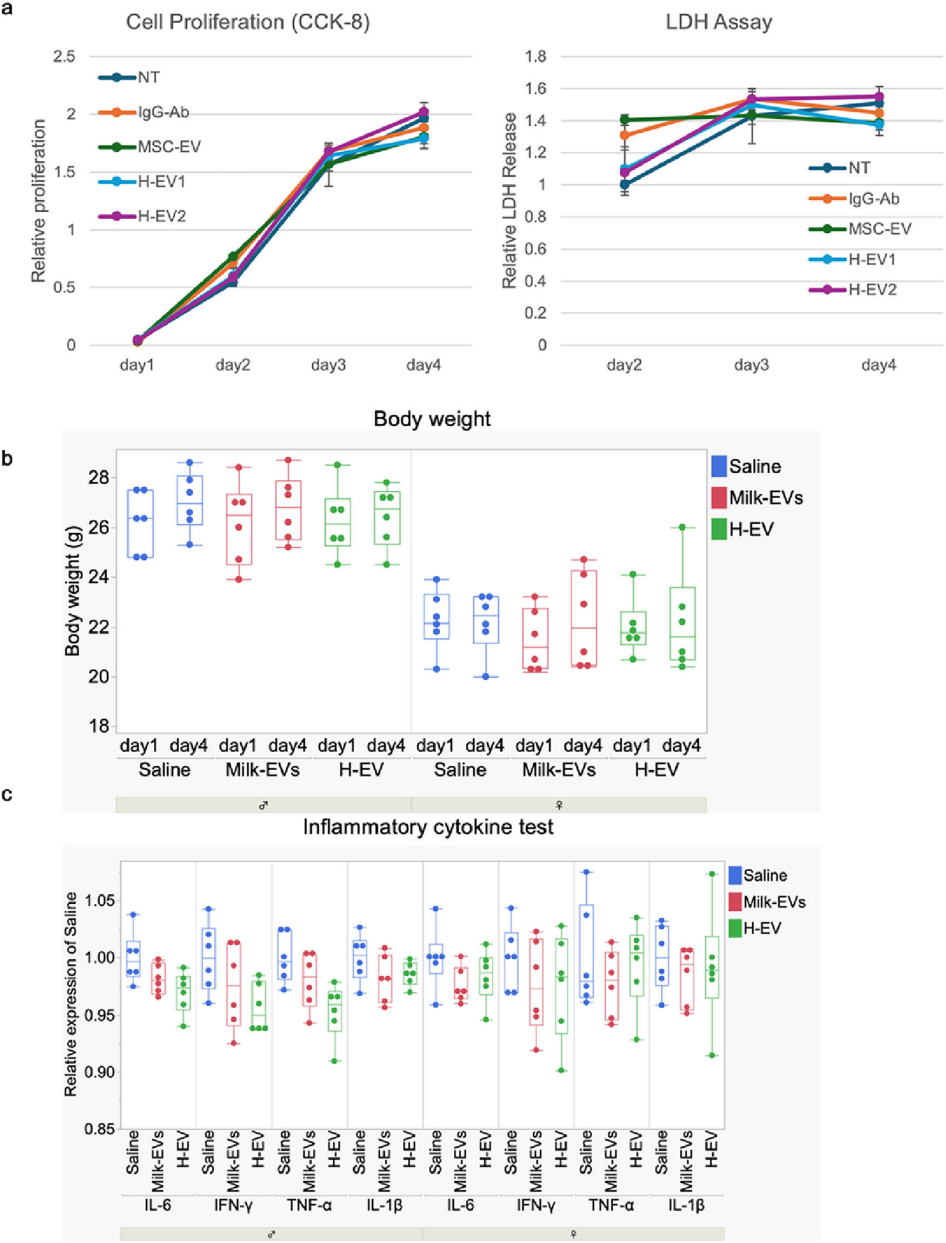
In vitro and In vivo toxicity of H‐EV. (a) Cell proliferation (CCK‐8 assay, left) and lactate dehydrogenase (LDH assay, right) in the culture medium were measured in HeLa cells monitored for 4 days with the following treatments: NT, IgG Abs, milk‐EVs alone, H‐EV1 and H‐EV2. *n* = 3, mean ± SD. (b) Changes in body weight (BW) of male (left) and female (right) mice 4 days after a single intravenous injection of saline (200 µL), milk‐EVs alone (1 mg/kg BW) or Vivo H‐EV (Ab, 10 mg/kg, EV, 1 mg/kg). *n* = 6, mean ± SD. (c) Serum inflammatory cytokines (IL‐6, IFN‐γ, TNF‐α and IL‐1β) measured by ELISA following euthenasia on Day 4. Left: male, right: female, *n* = 6, mean ± SD. Ab, antibody; BW, body weight; EV, extracellular vesicle; H‐EV, hybrid‐EV.

Some DDS materials may exhibit cytotoxicity, which limits their use in cells and in vivo. We evaluated the cytotoxicity of H‐EV in vitro by assessing cdll proliferation and membrane integrity. For the in vitro study, HeLa cells were untreated (NT) or treated with IgG Abs alone (200 ng), MSC‐EVs alone (200 ng), H‐EV1 or H‐EV2, and then cytotoxicity was monitored until Day 4 (Figure 8a). Cell proliferation was assessed using the CCK‐8 assay. The LDH assay was used to measure membrane damage‐induced LDH release as an indicator of cytotoxicity. The results showed that in terms of proliferative capacity, H‐EV1 and H‐EV2 had no effect on cell proliferation compared with that of the NT, IgG Abs alone and MSC‐EVs alone groups up to Day 4. For the LDH release experiment, the LDH signal of the NT group at Day 2 was set to 1 as a reference. At Day 2, both the IgG Abs alone and MSC‐EVs alone groups showed a minor but not statistically significant increase in LDH activity compared with that of the NT group. However, H‐EV1 and H‐EV2 exhibited LDH activity comparable to that of the NT group. By Days 3 and 4, the LDH activity of the H‐EV1 and H‐EV2 groups showed a slight increase relative to that at Day 2 but still remained at a level comparable to that of the NT group. Similarly, the IgG Abs alone and MSC‐EVs alone groups also maintained LDH levels comparable to that of the NT group at Days 3 and 4. These findings indicate that H‐EV encapsulating Abs exert minimal effects on both cell proliferation and cytotoxicity, suggesting favourable biocompatibility under the tested conditions.

### 3.7 Toxicity of Vivo H‐EV in Mice

Vivo H‐EVs (IgG Abs: 10 mg/kg BW, milk‐EVs: 1 mg/kg BW) were intravenously administered to 8‐week‐old male and female mice. Blood samples were collected 72 h post‐administration to evaluate acute toxicity following a single intravenous dose. As shown in Figure 8b, no significant changes in BW were observed in either male (*n* = 6) or female (*n* = 6) mice of the Vivo H‐EV group compared with those of the milk‐EVs‐only and saline groups before and 72 h after administration. Furthermore, as shown in Table S2, general blood tests revealed no significant changes in WBC, RBC, HGB, HCT, MCV, MCH, MCHC or PLT levels in the Vivo H‐EV group compared with those in the saline and milk‐EVs‐only groups in both male and female mice. Similarly, as presented in Table S3, liver function markers (AST, ALT and T‐BIL) and kidney function markers (BUN and CRE) did not show any significant differences between the groups. However, MCHC levels in male mice showed a statistically significant decrease (*p* < 0.05) in the Vivo H‐EV group compared with that of the saline group, and CRE levels in female mice showed a significant decrease (*p* < 0.05) in the Vivo H‐EV group compared with that in the saline group. Levels of inflammatory cytokines, including IL‐6, IFN‐γ, TNF‐α and IL‐1β, were measured using a mouse pro‐inflammatory cytokine multiplex kit (Figure 8c). Compared with saline controls, no significant changes were observed in the inflammatory cytokine levels of the milk‐EVs‐only or Vivo H‐EV groups. These results confirm that, within the administered dose range, Vivo H‐EV exhibited no toxicity in both cellular and small animal models, indicating its biocompatibility.

## 4 Discussion

In this study, we employed an ILB composed of EDMPC and linoleate to enable efficient post‐loading of macromolecular cargo into EVs. EDMPC is a permanently cationised derivative of DMPC, a zwitterionic phosphatidylcholine widely used in model membrane studies because of its low phase transition temperature and high membrane fluidity. Unlike native DMPC, which is electrically neutral under physiological conditions and exhibits limited fusogenicity, EDMPC provides a stable positive charge that promotes electrostatic interaction and fusion with negatively charged EV membranes. Structurally, EDMPC retains high homology to endogenous phospholipids compared with synthetic cationic lipids such as DOTAP or DOPE, which may potentially reducing metabolic and biocompatibility concerns. Linoleate, a naturally occurring unsaturated fatty acid, was used as a counterion to loosen lipid packing and further facilitate membrane fusion, thereby potentially enhancing intracellular delivery efficiency.

Current limitations of EV‐based DDSs include low encapsulation efficiency, poor molecular selectivity and challenges in achieving non‐disruptive and uniform particle formulations. The ILB used in this study forms vesicle structures ranging from approximately 80 to 150 nm in size by binding with nucleic acids, peptides or proteins, and exhibits high fusogenicity with lipid bilayer structures present in cells and other carriers. These characteristics motivated us to investigate the interaction between ILB and EV membranes due to their intrinsic cell‐derived proterties and delivery potential. For instance, EVs secreted from neurodegenerative cells transport αSyn to other neural cells (Kara et al. 2018). Because EVs typically carry a negative charge of approximately −10 to −20 mV, we hypothesised that positively charged particles composed of ILB and molecular complexes would electrostatically interact and fuse with H‐EV.

EVs were isolated from bovine milk and MSC cultures in accordance with the MISEV guidelines. Indeed, primary particles composed of ILB and target molecules, as well as H‐EV using various EVs types, were prepared, thereby establishing a post‐loading technique for EVs. A reproducible protocol for the formation of H‐EV with different target molecules was developed. Importantly, H‐EVs prepared from MS‐EVs isolated at different time points and passages exhibited comparable particle size distributions and surface charges, indicating good batch‐to‐batch reproducibility of the hybridisation process (Figure S10). FCM analysis confirmed the complexation of H‐EV, as indicated by a fluorescence shift of the original EVs population after H‐EV preparation. However, FCM analysis alone could not determine whether the loaded Abs or nucleic acids were encapsulated inside the EVs or merely bound to their lipid membranes. To address this, we conducted enzymatic degradation experiments using proteinase K and Triton X‐100 on H‐EV loaded with the model molecule αSyn Ab and milk‐EVs. WB analysis revealed that αSyn Abs remained intact after enzymatic treatment, indicating that the Abs were protected within the EVs. Furthermore, when EV membranes were perforated with Triton X‐100 and treated with proteinase K, all Ab signals were eliminated, confirming that the Abs had indeed been encapsulated. Similarly, nucleic acids encapsulated in H‐EV were verified using RNase treatment and electrophoresis. These findings suggest that ILB‐complexed αSyn Abs and miRNA were incorporated within EVs and protected by the EVs membrane. Although these protection assays strongly support membrane fusion‐mediated encapsulation rather than simple surface adsorption, direct quantification of membrane fusion efficiency using lipid‐ or content‐mixing assays remains an important subject for future studies.

In addition to qualitative encapsulation analyses, we quantitatively compared the loading efficiency of our ILB‐based H‐EV method with commonly used post‐loading approaches, including electroporation, freeze–thaw cycling and simple incubation (Figure S7). The ILB‐mediated hybridisation strategy consistently demonstrated higher encapsulation efficiency for both Abs and nucleic acids than the conventional methods. Electroporation, while widely used, is known to induce EV membrane disruption, cargo aggregation and heterogeneous particle populations, which can limit effective encapsulation. Similarly, freeze–thaw and passive incubation methods rely primarily on transient membrane permeability or surface adsorption, often resulting in low loading efficiency and poor reproducibility. In contrast, the ILB‐based approach enables electrostatically driven complex formation followed by membrane fusion, allowing efficient incorporation of macromolecular cargo into EVs while preserving EV integrity. These comparative results highlight a key advantage of the H‐EV strategy over existing post‐loading techniques and underscore its potential as a robust and scalable platform for intracellular delivery of therapeutic proteins and nucleic acids.

Nucleic acids are highly susceptible to enzymatic degradation. When administered in vivo, nucleic acids are rapidly degraded by bloodborne enzymes, leaving only a minimal fraction that reaches the target site. In contrast, Abs are relatively stable in the bloodstream. Abs circulate in the bloodstream until they find their target but struggle to penetrate deep into organs with barrier functions. Numerous DDS technologies, such as LNPs for nucleic acid delivery, have been developed, but challenges remain, including toxicity and inefficient endosomal escape (Chatterjee et al. 2024). The use of ILB to enable the encapsulation of nucleic acids and Abs within natural DDS materials such as EVs has the potential to overcome these challenges. EVs uptake by cells occur through multiple pathways, including membrane fusion (Del Conde et al. 2005), macropinocytosis (Tian et al. 2014; Nakase et al. 2015) and endocytosis (Mathieu et al. 2019; McKelvey et al. 2015). We investigated the uptake mechanism of IgG Ab‐loaded H‐EV (derived from milk) using endosomal inhibitors and found that H‐EV primarily enter cells via macropinocytosis. This indicates that H‐EVs are internalised through the same mechanisms as native EVs.

Next, we evaluated the functionality of Abs that escaped from endosomes into the cytoplasm using two distinct in vitro model systems. First, we evaluated whether MSC‐derived H‐EV loaded with αSyn Abs and introduced simultaneously with αSyn aggregates could bind to intracellular antigens and inhibit αSyn aggregation. The results showed that although αSyn Abs alone did not completely prevent αSyn aggregation, the H‐EV group exhibited a significant suppression of αSyn aggregation within cells. Previous studies have reported that MSC‐EVs derived from umbilical cord mesenchymal stem cells can suppress αSyn aggregation in an in vitro Parkinson's disease model (Chen et al. 2020). However, in our study, MSC‐EVs derived from adipose‐derived mesenchymal stem cells did not exhibit such inhibitory effects on αSyn aggregation. This discrepancy is likely attributable to differences in the endogenous components depending on the cell source of the EVs. These results suggest that the therapeutic or prophylactic utility of EVs in drug delivery could be enhanced by encapsulating specific bioactive molecules. Anti‐αSyn Abs bind to the C‐terminus of extracellular αSyn proteins, and the resulting complex is degraded by lysosomes (George and Brundin 2015). It is likely that the same mechanism is responsible for the binding and degradation of intracellular proteins. Similarly, in an apoptosis‐inducing model targeting AKT protein, we confirmed that MSC‐EVs encapsulating pAKT Abs activated caspase‐3/7. Previous studies have reported that encapsulating pAKT Abs in LNPs and delivering them intracellularly suppressed AKT phosphorylation, leading to increased caspase‐3/7 activity (Hirai et al. 2024). Interestingly, our approach achieved comparable caspase‐3/7 activation at a lower Ab concentration than previous LNP‐based methods (Hirai et al. 2024).

Importantly, the utility of our H‐EV technology was also demonstrated in vivo. Fluorescently labelled IgG Abs encapsulated in EVs were detected within cells in the cerebral cortex, cerebellum and hippocampus following intravenous administration, suggesting successful passage across the BBB. Consistent with a non‐disruptive loading process, H‐EVs retained canonical EV surface markers and intact bilayer morphology without apparent aggregation, as confirmed by WB and TEM analyses. Further studies are underway to quantitatively assess the proportion of administered Abs delivered to specific brain cell types and to enhance targeting specificity and delivery efficiency. Our approach represents a novel DDS strategy with considerable therapeutic potential for the treatment of neurodegenerative diseases, where traditional methods possess limited ability to transport macromolecules across the BBB.

Regarding clinical translation, the scalability of H‐EV production is an important consideration. The current preparation method is based on simple mixing and self‐assembly processes, which may be adaptable to large‐scale manufacturing. Although this study was conducted at a laboratory scale, future optimisation towards GMP‐compliant production will be required. From a regulatory perspective, H‐EVs represent a novel drug delivery platform. Regulatory frameworks established for EVs, LNPs and Ab‐based therapeutics may provide a foundation; however, comprehensive evaluation of quality, safety and consistency will be necessary for clinical application. In terms of cost‐effectiveness, the H‐EV preparation process does not require genetic modification or complex chemical conjugation, which may offer advantages over existing Ab delivery systems. Nevertheless, detailed cost analyses will be needed in future translational studies.

## 5 Conclusion

A DDS strategy using our proprietary ILB to encapsulate nucleic acids and proteins within EVs was developed. The encapsulated active components demonstrated efficient cellular uptake, functionality, transportability and biocompatibility in both in vitro and in vivo evaluation systems. EVs represent a versatile and well‐established carrier platform with distinct characteristics depending on their cellular origin. Our innovative approach using ILB to incorporate therapeutic molecules into EVs enables the efficient loading of large bioactive molecules while preserving EVs integrity. This approach advances EVs‐based DDS and provides a novel strategy for targeted delivery of nucleic acid and Ab therapeutics. Although further optimisation, including quantitative fusion efficiency assessment and scalable purification strategies, will be required for clinical translation, currently, we are further optimising EVs containing therapeutics tailored to target specific diseases.

## Author Contributions

L.C. and K.I. designed the experiments. F.O., T.I. and M.G. synthesised ILB and performed the analysis. L.C. conducted cell treatments and animal experiments. L.C. and K.I. analysed the data. M.G. supervised the study. L.C., F.O. and K.I. wrote the manuscript.

## Conflicts of Interest

Kohei Ishihama is the founder and shareholder of NOVIGO Pharma Inc. Lin Cui, Fumiyasu Ono and Tomoko Ichinose are employees of the company. The other authors declare no conflicts of interest.

## Supporting information




**Supporting Information**: jex270125‐sup‐0001‐Figures.docx

**Supporting Information**: jex270125‐sup‐0002‐Tables.xlsx

## Data Availability

The data that support the findings of this study are available from the corresponding author upon reasonable request.
