# Supplementary material for: Macromolecule‐Loaded Hybrid Extracellular Vesicles via Ionic Lipid–Based Post‐Loading for Intracellular Delivery: Functional Evaluation for Neurodegenerative Therapy
Source: J Extracell Biol. 2026 Mar 17;5(3):e70125. doi: 10.1002/jex2.70125 (PMC13097689; doi:10.1002/jex2.70125)
Supplement: Supplementary file 1 — Supporting Information: jex270125‐sup‐0001‐Figures.docx [file JEX2-5-e70125-s002.docx]

Supplemental Figure 1


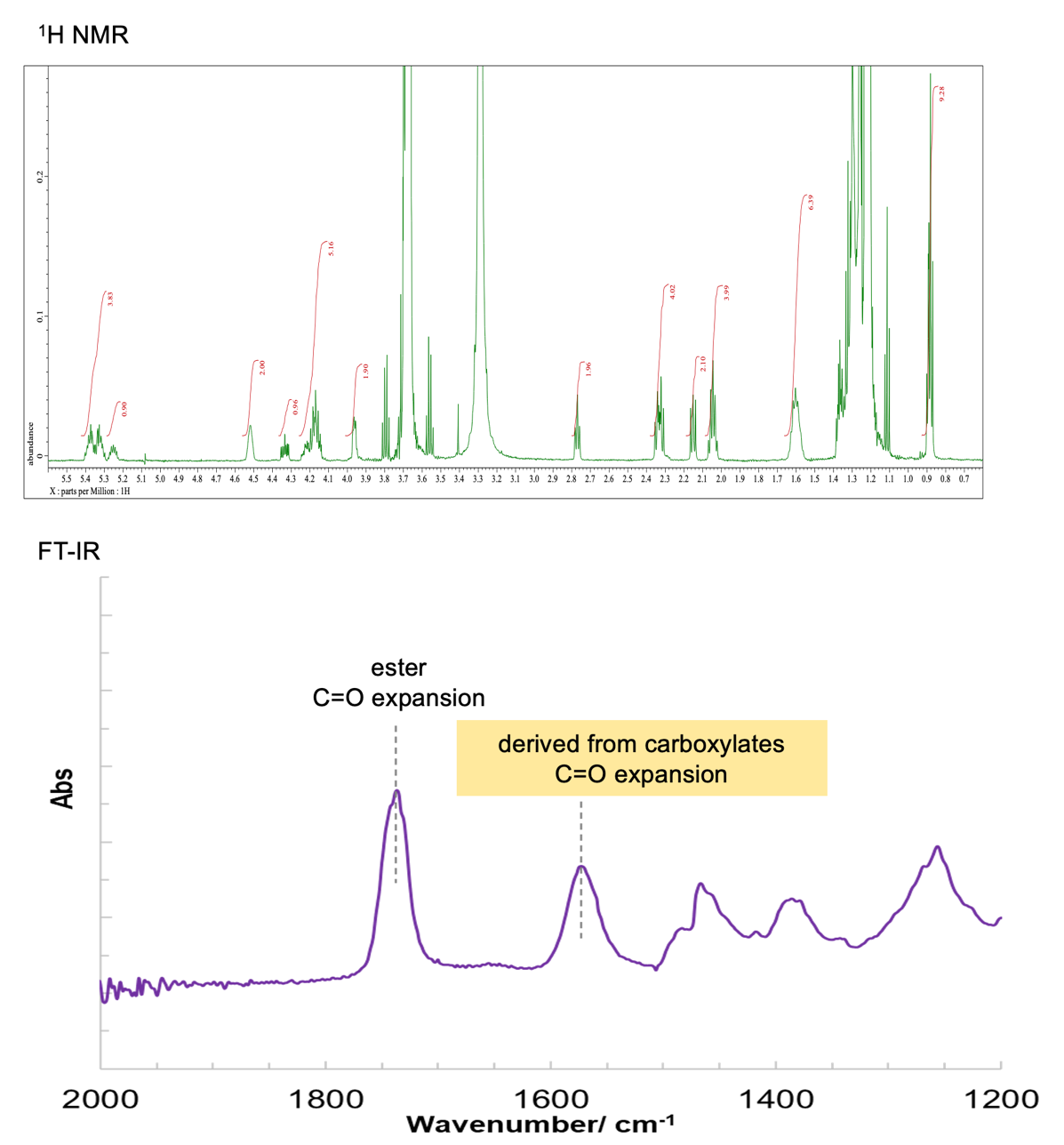


Supplemental Figure 2


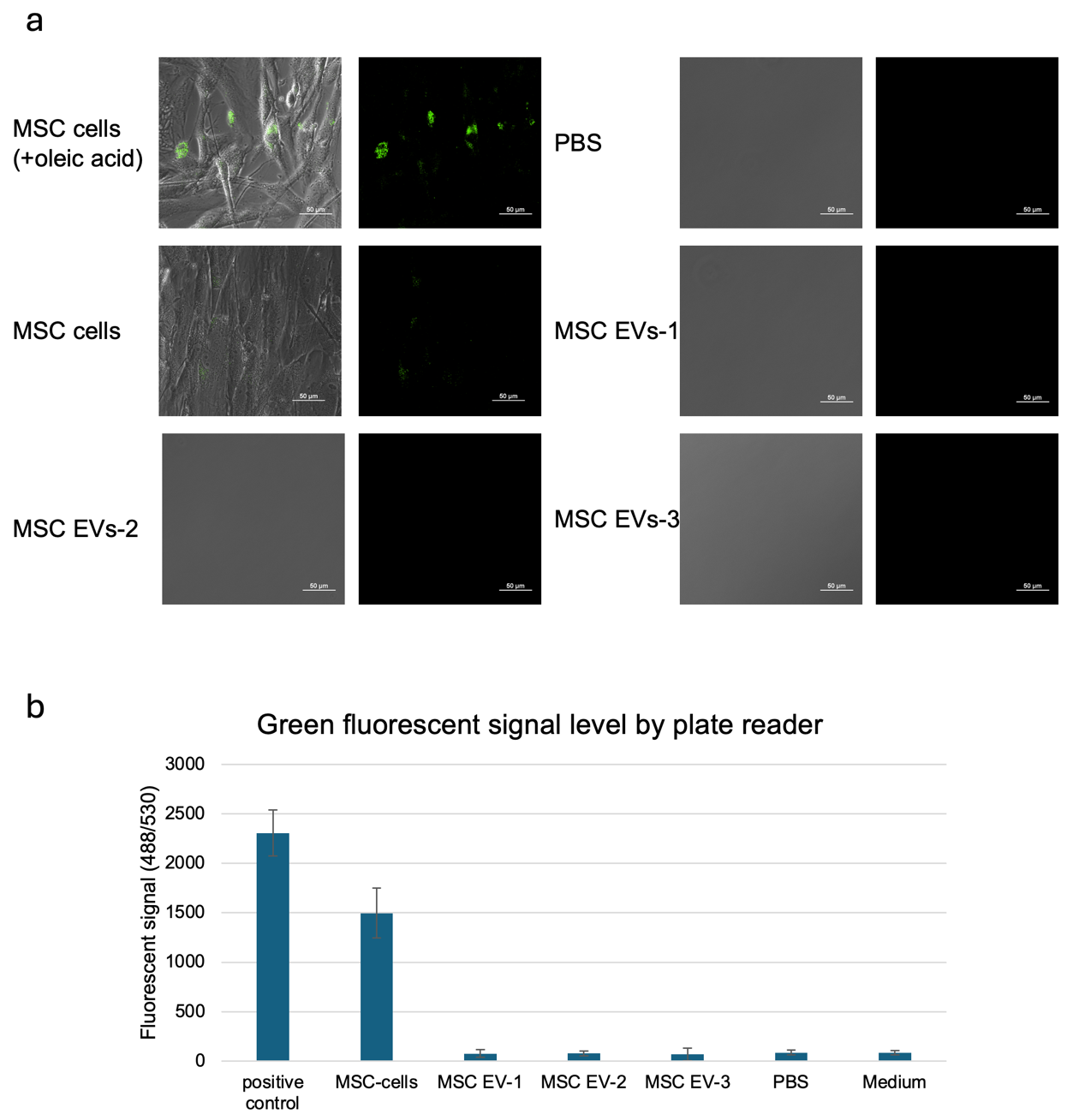


Supplemental Figure 3


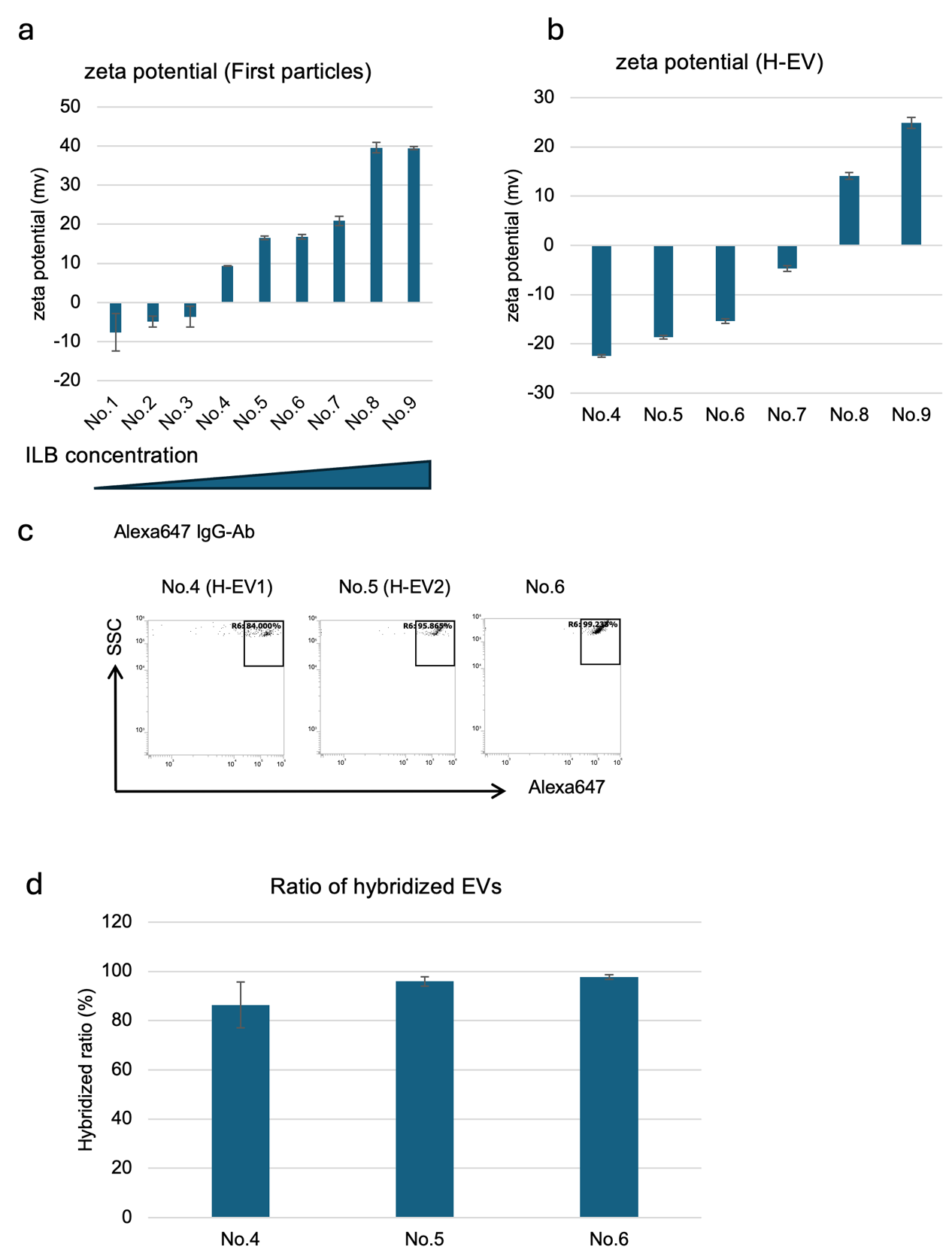


Supplemental Figure 4


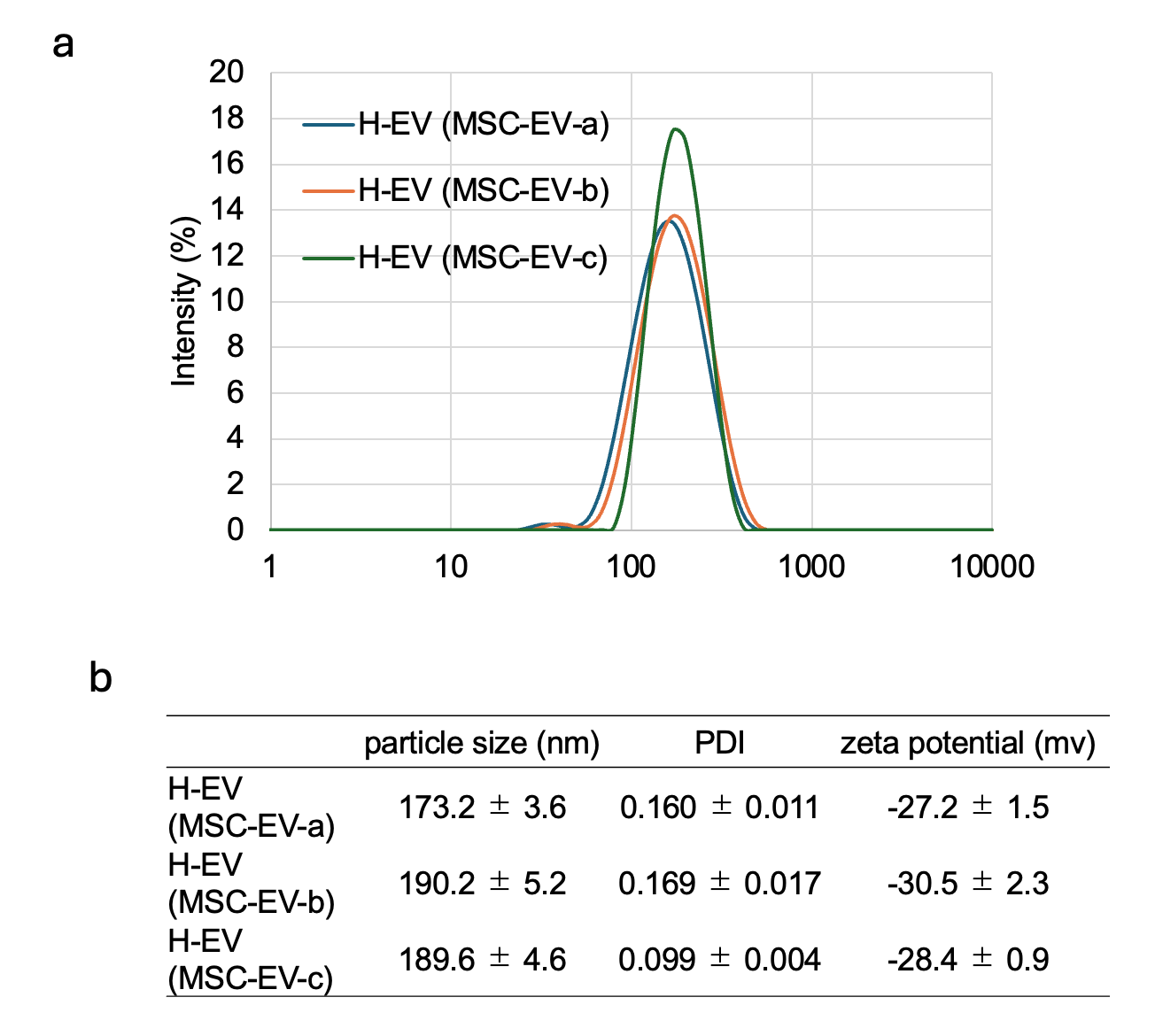


Supplemental Figure 5


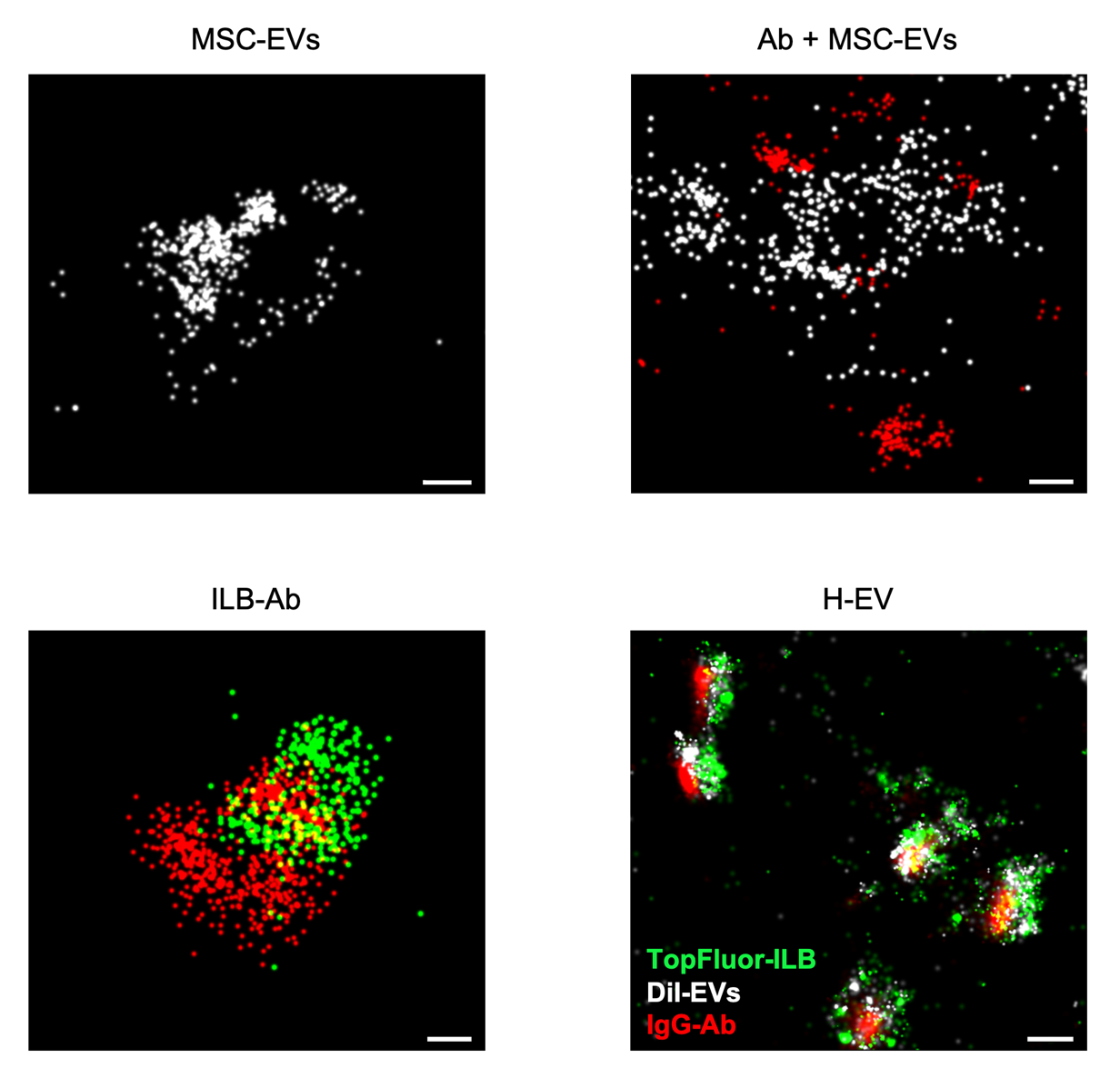


Supplemental Figure 6


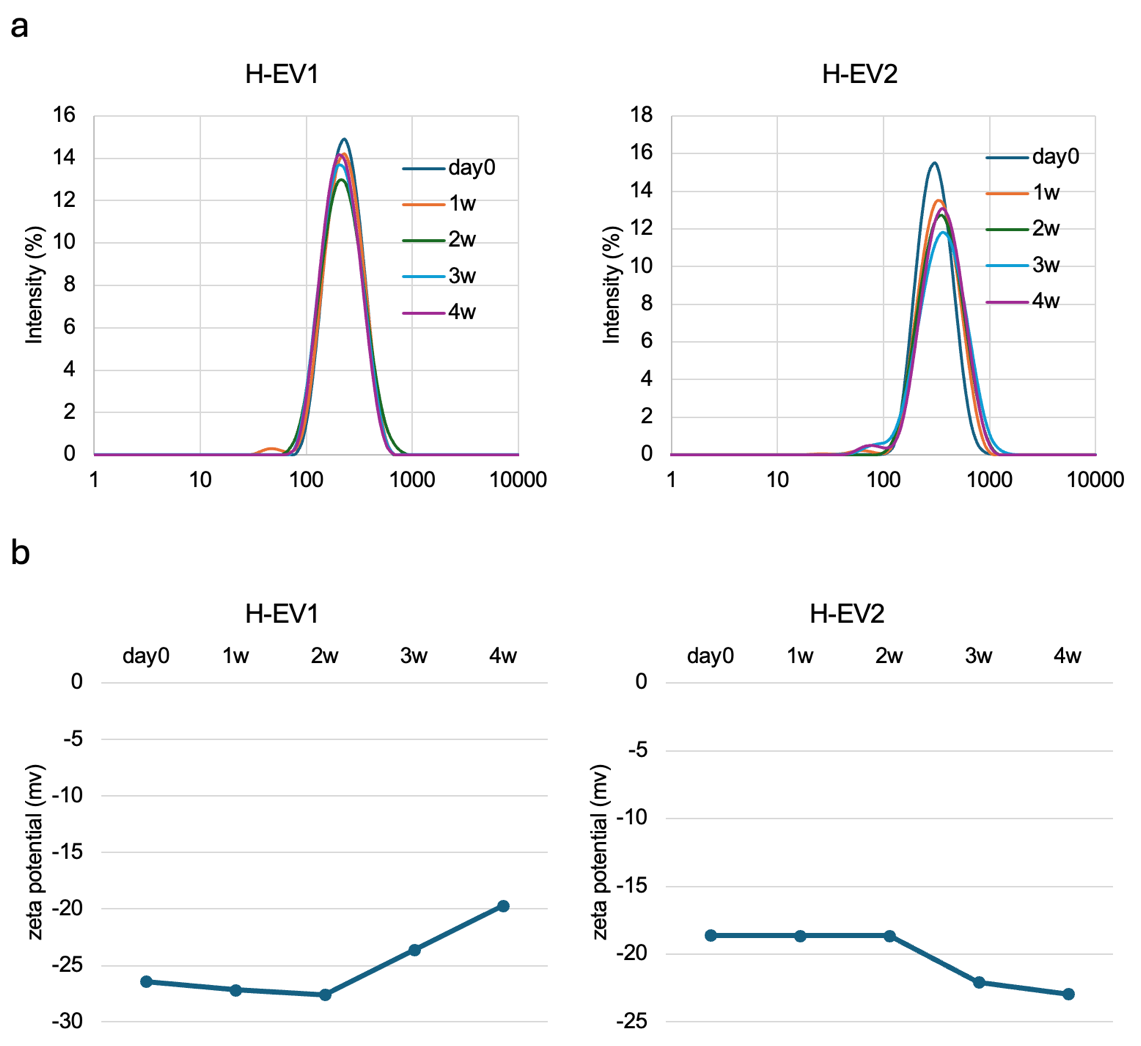


Supplemental Figure 7


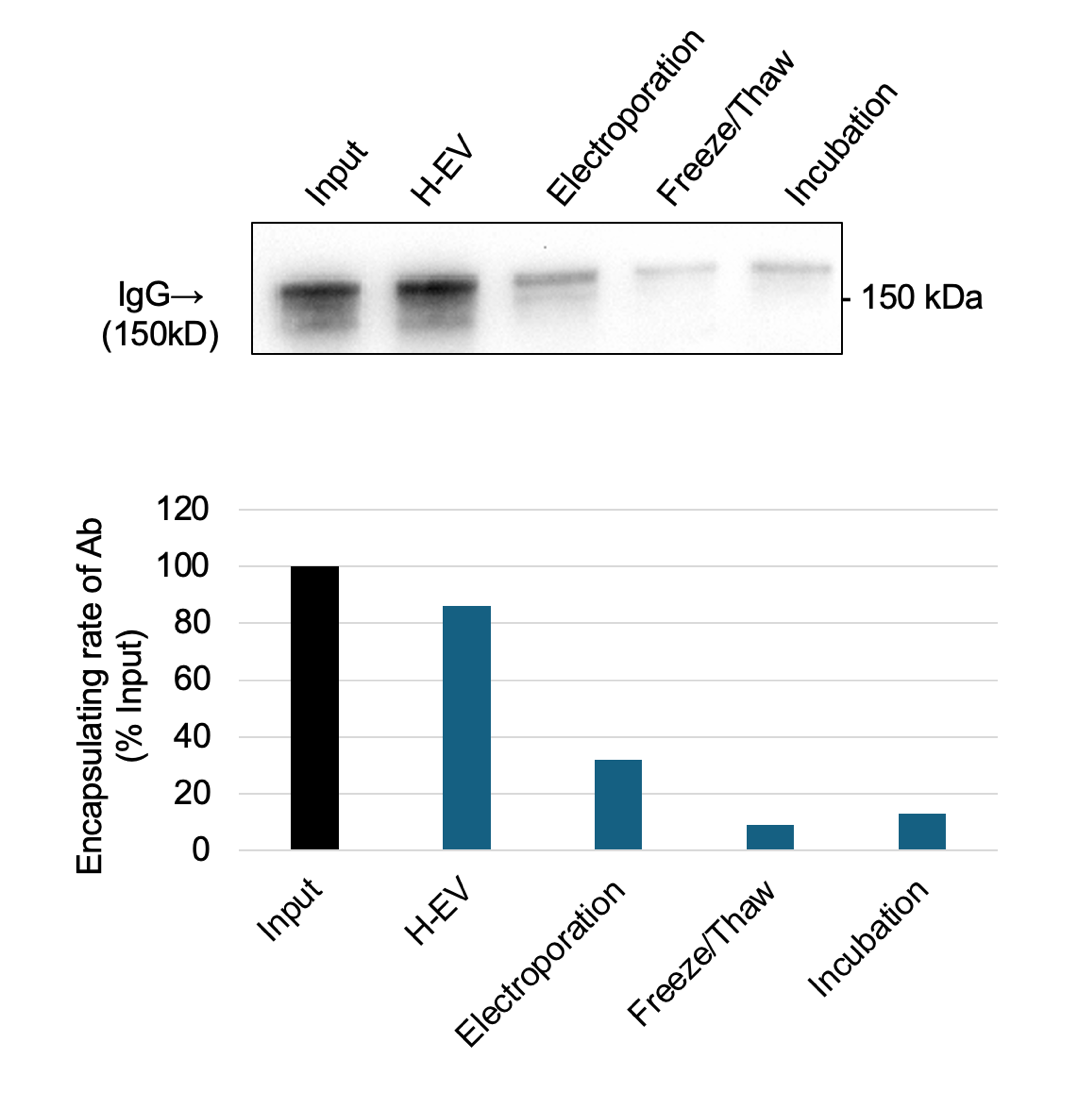


Supplemental Figure 8


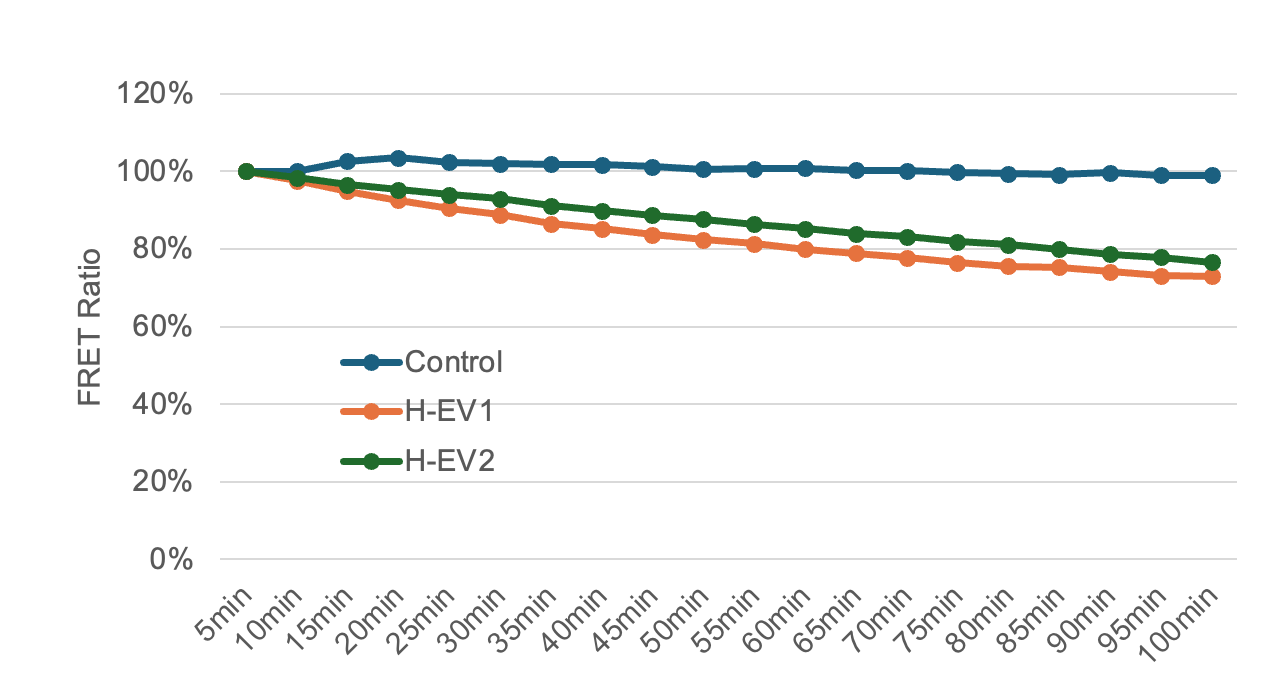


Supplemental Figure 9


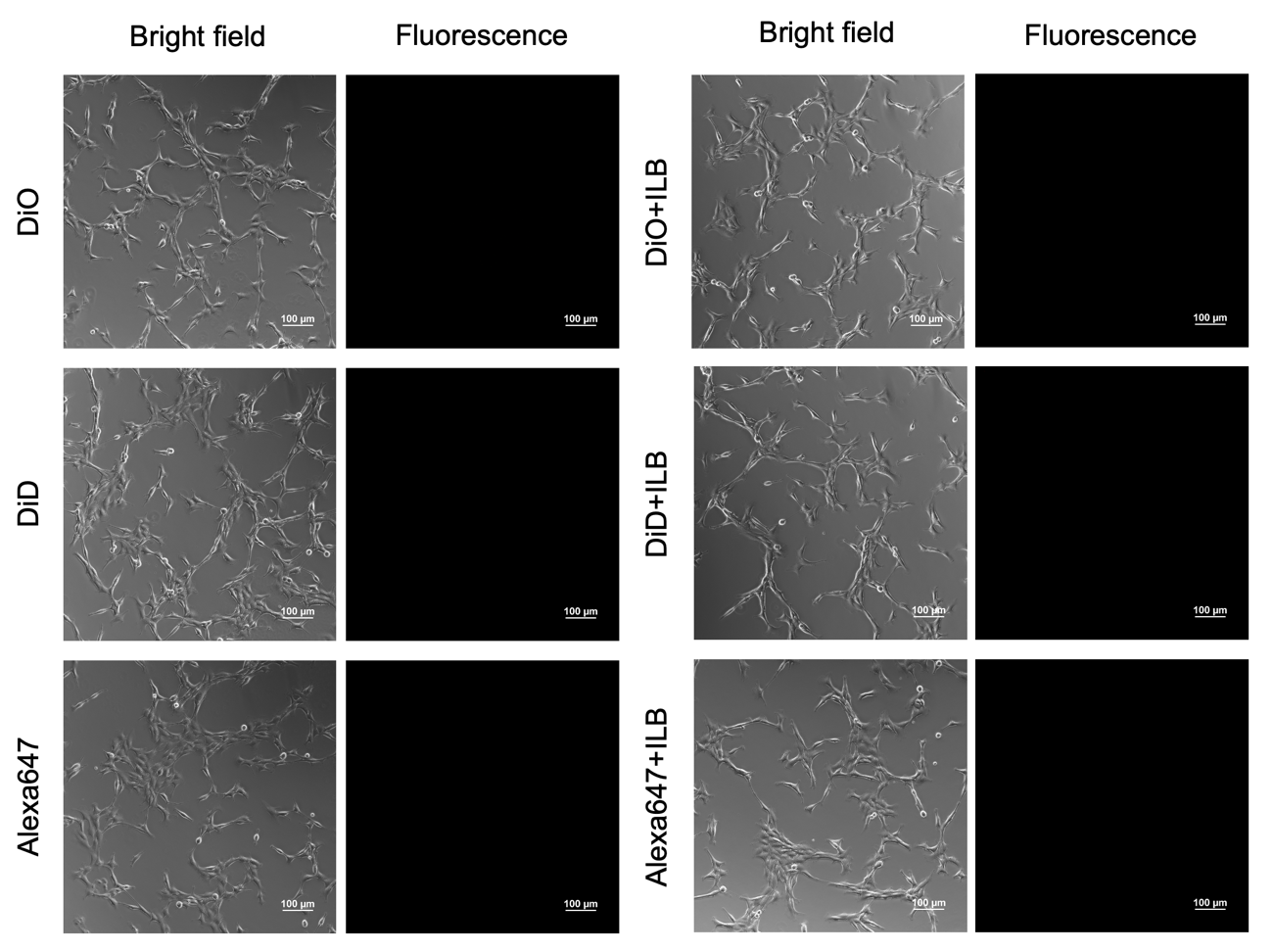


Supplemental Figure 10


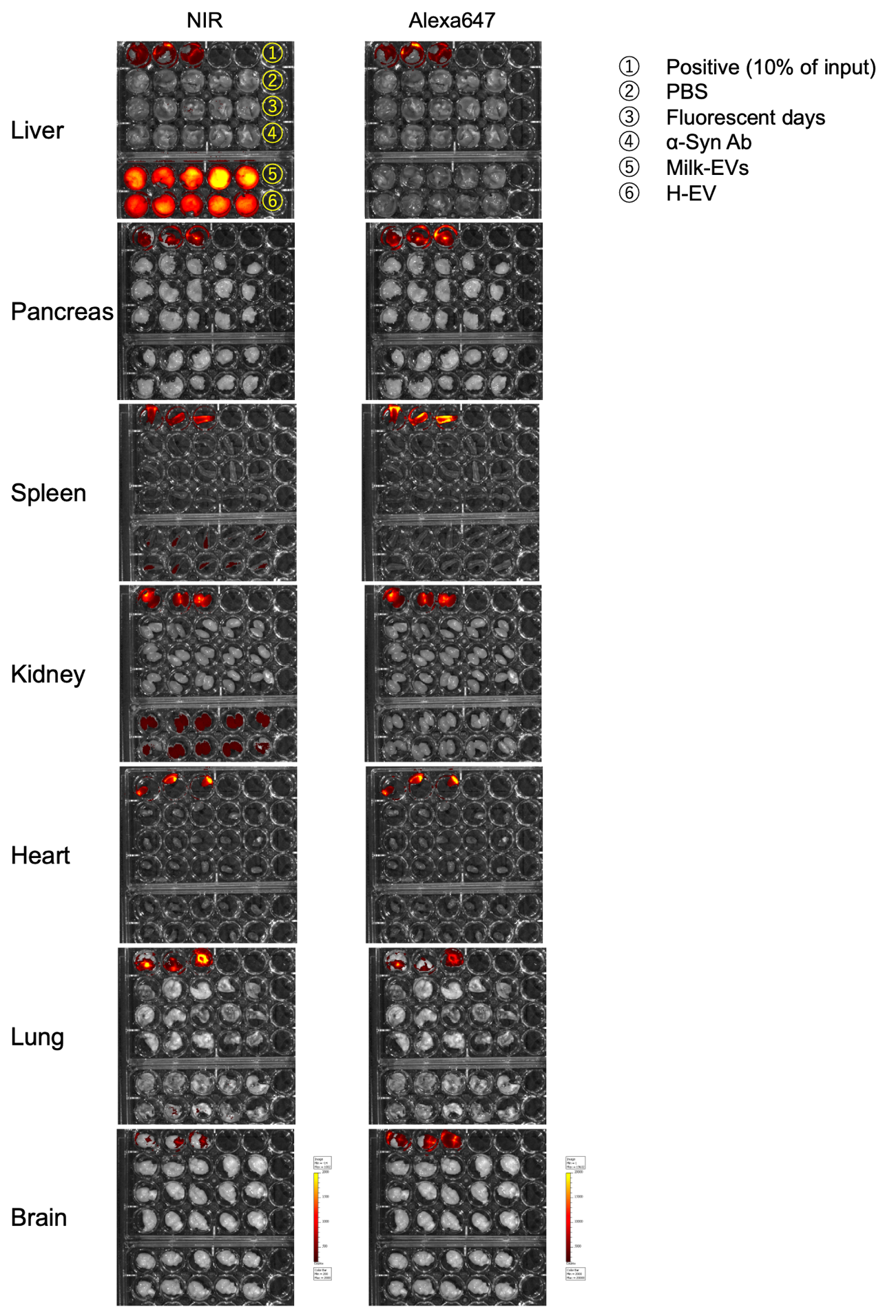


**List of Supplemental Figure Captions**

Supplemental Figure 1. ^1^H NMR and FT-IR analysis of ILB.

^1^H NMR and FT-IR spectra confirm the chemical structure of ILB. The ^1^H NMR spectrum shows proton signals consistent with the phosphatidylcholine framework and fatty acid chains. FT-IR analysis reveals characteristic ester C=O stretching and carboxylate-derived C=O signals, indicating successful formation of ILB composed of EDMPC and linoleic acid.

Supplemental Figure 2. Evaluation of lipid droplet contamination in MSC-derived EV preparations. (a) Fluorescence microscopy images of human adipose-derived mesenchymal stem cells (MSCs) and MSC-derived EVs stained with Lipi-Green dye. Oleic acid–treated MSCs (+ oleic acid) showed strong intracellular green fluorescence, indicating abundant lipid droplet formation. In contrast, untreated MSCs exhibited weak fluorescence. No detectable Lipi-Green fluorescence was observed in MSC-derived EV samples (MSC EV-1, MSC EV-2, MSC EV-3) or PBS controls, indicating the absence of lipid droplet contamination in isolated EV fractions. Scale bars, 50 μm. (b) Quantification of Lipi-Green fluorescence intensity measured by a plate reader. Oleic acid–treated MSCs (positive control) showed high fluorescence signals, whereas untreated MSCs displayed moderate fluorescence. In contrast, MSC-derived EV samples (MSC EV-1, MSC EV-2, MSC EV-3), PBS, and culture medium showed only background-level signals. Data are presented as mean ± SD (n = 3 independent experiments).

Supplemental Figure 3. Optimization of the mixing ratio of ILB, antibody, and EVs for H-EV preparation. (a) Zeta potential of ILB–IgG primary particles prepared with increasing ILB concentrations (No.1–No.9). As the ILB ratio increased, the surface charge of the primary particles gradually shifted from negative to positive, with particles from No.4 onward exhibiting a net positive charge. (b) Zeta potential of hybrid EVs (H-EVs) generated by mixing EVs with ILB–IgG primary particles (No.4–No.9). H-EVs prepared using No.4–No.6 retained a net negative surface charge comparable to native EVs, whereas No.7 approached neutral charge and No.8–No.9 became positively charged, indicating excessive ILB incorporation. (c) Flow cytometry (FCM) analysis of Alexa Fluor 647–labeled IgG antibody loading into H-EVs prepared using ILB–IgG primary particles No.4–No.6. Representative dot plots show Alexa Fluor 647–positive EV populations. (d) Quantification of the ratio of hybridized EVs based on the proportion of Alexa Fluor 647–positive events detected by FCM. H-EVs prepared using No.4–No.6 exhibited high hybridization efficiency. Based on comparable size distribution, surface charge, and encapsulation efficiency between No.5 and No.6, No.4 and No.5 were selected as H-EV1 and H-EV2, respectively, to minimize ILB content while maintaining optimal performance. Data are presented as mean ± SD (n = 3 independent experiments).

Supplementary Figure 4. Reproducibility of H-EV preparation using MSC-EVs derived from independent donors. Hybrid EVs (H-EV) were prepared using EVs derived from three independent mesenchymal stem cell (MSC) cultures (MSC-EV-a, MSC-EV-b, and MSC-EV-c) under identical preparation conditions. (a) Particle size distributions of the resulting H-EVs were analyzed by DLS, showing comparable size profiles among the three preparations. (b) The mean particle size, polydispersity index (PDI), and zeta potential of each H-EV preparation are summarized. No significant differences were observed among H-EV generated from different MSC-EVs sources, indicating minimal donor-to-donor variability and high reproducibility of the H-EV preparation method.

Supplementary Figure 5. Single-molecule localization microscopy (SMLM) imaging of hybrid EV formation. Single-molecule localization microscopy (SMLM) images acquired using a Nanoimager system showing the spatial distribution of EVs, antibodies, and ILB in different sample conditions. Top left: MSC-derived EVs (DiI-labeled; white). Top right: Mixture of Alexa Fluor 647–labeled IgG antibodies (red) and MSC-EVs without ILB, showing minimal colocalization. Bottom left: ILB–Ab primary particles labeled with TopFluor (green, ILB) and Alexa Fluor 647 (red, IgG), demonstrating the formation of ILB–Ab complexes. Bottom right: Hybrid EVs (H-EV) generated by mixing ILB–Ab primary particles with EVs, showing clear nanoscale colocalization of EV membranes (DiI, white), ILB (TopFluor, green), and IgG antibodies (Alexa Fluor 647, red). (scale bar = 100 nm)

Supplemental figure 6. Long-term stability of H-EVs stored at room temperature.

Long-term stability of H-EV1 and H-EV2 stored under room temperature conditions was evaluated for up to 4 weeks. (a) Particle size distribution profiles of H-EV1 and H-EV2 measured by DLS at day 0 and after 1, 2, 3, and 4 weeks of storage. No substantial changes in size distribution were observed over the storage period. (b) Zeta potential of H-EV1 and H-EV2 measured at the indicated time points. Although minor fluctuations were observed, the overall surface charge remained relatively stable throughout the 4-week storage period. These results indicate that H-EVs maintain their physicochemical stability during prolonged storage at room temperature.

Supplementary Figure 7. Comparison of antibody encapsulation efficiency among different EV loading methods.

Comparison of IgG antibody encapsulation efficiency into EVs using different loading methods. Upper panel shows representative Western blot images detecting IgG (150 kDa) in input samples and EVs prepared by H-EV formation, electroporation, freeze–thaw cycling, or simple incubation. Equal protein amounts were loaded for each condition.

Lower panel shows quantitative analysis of encapsulation efficiency, expressed as the percentage of IgG signal relative to the input. The H-EV method achieved substantially higher encapsulation efficiency compared with electroporation, freeze–thaw, and incubation methods.

Supplementary Figure 8. FRET analysis demonstrating nanoscale interaction between ILB–cargo complexes and EV membranes.

Förster resonance energy transfer (FRET) analysis was performed to examine the physical association between differentially labeled ILB–cargo complexes and EV membranes.

Time-dependent changes i

n the FRET ratio were monitored for control samples, H-EV1, and H-EV2 over 100 min.

Supplementary Figure 9. Cellular uptake of fluorescent dyes alone or ILB–dye complexes.

Representative bright-field and fluorescence images of cells treated with fluorescent dyes alone or dye–ILB complexes. Cells were incubated with DiO, DiD, or Alexa Fluor 647 either alone or pre-mixed with ILB under the same conditions used for H-EV preparation. Bright-field images show normal cell morphology, whereas fluorescence images reveal no detectable intracellular fluorescence signal in any condition. Scale bar: 100 µm.

Supplemental Figure 10. Organ distribution of H-EVs evaluated by IVIS imaging.

Representative IVIS images showing the organ distribution of Alexa Fluor 647–labeled antibodies (Alexa647-Ab) in major organs 24 h after intravenous administration. ① Quantification standard: 10% of the total injected dose was directly added to each excised organ to generate a reference signal for normalization. ② PBS control group. ③Dye-only control group receiving free NIR dye and Alexa Fluor 647 dye at amounts equivalent to those used for labeling. ④α-synuclein antibody alone (α-Syn Ab) group. ⑤ Milk-derived EV (milk-EV) group. ⑥ Hybrid EV (H-EV) group. All formulations were diluted in physiological saline and administered intravenously to mice. Twenty-four hours after injection, mice were sacrificed, perfused with PBS followed by 4% PFA, and major organs were excised and imaged using an IVIS imaging system. The fluorescence intensity in each organ was used to evaluate the biodistribution of Alexa647-Ab.
